# Behavioral responses by an apex predator to urbanization

**DOI:** 10.1093/beheco/arz019

**Published:** 2019-03-02

**Authors:** E Hance Ellington, Stanley D Gehrt

**Affiliations:** School of Environment and Natural Resources, Ohio State University, Columbus, OH, USA

**Keywords:** Chicago, coyote (*Canis latrans*), foraging, home range, movement behavior

## Abstract

Wildlife can respond to urbanization positively (synanthropic) or negatively (misanthropic), and for some species, this is a nonlinear process, whereby low levels of urbanization elicit a positive response, but this response becomes negative at high levels of urbanization. We applied concepts from foraging theory to predict positive and negative behavioral responses of coyotes (*Canis latrans*) along an urbanization gradient in the Chicago metropolitan area, USA. We estimated home range size and complexity, and metrics of 3 movement behaviors (encamped, foraging, and traveling) using Hidden Markov movement models. We found coyotes exhibited negative behavioral responses to highly urbanized landscapes: coyotes viewed the landscape as lower quality, riskier, and more fragmented (home range size and complexity, and time spent encamped increased). Conversely, we found evidence of both positive and negative responses to suburban landscapes: coyotes not only viewed the landscape as higher quality than natural fragments and equally risky, but also viewed it as fragmented (home range size decreased, time spent encamped did not change, and home range complexity increased). Although the spatial and behavioral responses of coyotes to urbanization became increasingly negative as urbanization increased, coyotes were still able to occupy highly urbanized landscapes. Our study demonstrates how wildlife behavioral responses can be dependent on the degree of urbanization and represents one of the first descriptions of apex predator space use and movement in a highly urbanized landscape.

## Teaser

As urbanization increases globally, wildlife must respond. We applied concepts from foraging behavior to determine how coyote movement behavior varies across a gradient of urbanization. In suburban landscapes, coyotes responded positively by requiring less space to live. In highly urbanized landscapes, coyotes responded negatively by requiring more space and spending more time not moving. Despite these challenges, coyotes can thrive in highly urbanized landscapes, such as downtown Chicago, USA.

## INTRODUCTION

Human population abundance and the extent of urban area have increased worldwide (Seto et al. 2011; United Nations 2014), and future growth is predicted to occur at unprecedented rates (Seto et al. 2012). Furthermore, urban expansion will probably continue to occur near protected areas and in areas of high biodiversity (McDonald et al. 2008; Seto et al. 2012). Conversion of the natural landscape into urban and suburban land, however, does not necessarily result in local declines in species richness. Rather, it can cause a shift in the assemblage of species in urban environments, where native species are occasionally replaced by non-native, urban-adapted species (McKinney 2006; Shochat et al. 2010).

Wildlife species can respond to urbanization in several ways. Examples of positive (synanthropic) responses include increased population density in urban relative to natural areas (Prange et al. 2003), utilization of anthropogenic resources such as garbage (Prange et al. 2004), and loss of fear near human presence (Kark et al. 2007). Conversely, examples of negative (misanthropic) responses include altering movement, shifting behavior both spatially and temporally to avoid high human activity or human-dominated land cover types (Mitchell et al. 2015; Poessel et al. 2016; Tucker et al. 2018). Some species can be classified as strongly synanthropic, such as the rock dove (*Columba livia*), brown rat (*Rattus norvegicus*), and several cockroach species (*Blattidae*), whereas others can be described as strongly misanthropic, such as tailed frogs (*Ascaphus truei*) and the spotted owl (*Strix occidentalis*). Most species, however, probably fall somewhere in between, whereby they can tolerate some urbanization but cannot persist in highly urbanized landscapes or can persist in urban environments but display negative responses to urbanization (e.g., high mortality, low density, and spatial and temporal avoidance of humans). A more complete understanding of how the spatial and behavioral response of wildlife species to urbanization might vary with the degree of urbanization would allow us to better predict the effects of urbanization on global biodiversity. We propose a mechanistic approach to understanding the relationship between urbanization and wildlife behavior based on foraging theory.

As urbanization increases, the quantity of natural landscape decreases and fragments (McKinney 2006), which could lead to a decrease in the availability and predictability of natural prey. Concurrently, an increase in human-associated landscapes leads to an increase in the availability and predictability of anthropogenically sourced food such as garbage (Oro et al. 2013). In addition, nonpermeable, human-associated features (e.g., large buildings) increase with urbanization (McKinney 2006), precluding most, if not all, use by terrestrial wildlife. Thus, in highly urbanized landscapes, both natural and permeable human-associated landscapes have fragmented and decreased in the landscape. Finally, as urbanization increases, human presence, regardless of landscape type, increases. The changes wrought by urbanization undoubtably affect wildlife species by altering what an individual perceives as a habitat patch, and the characteristics and quality of that patch.

**Figure 1:**
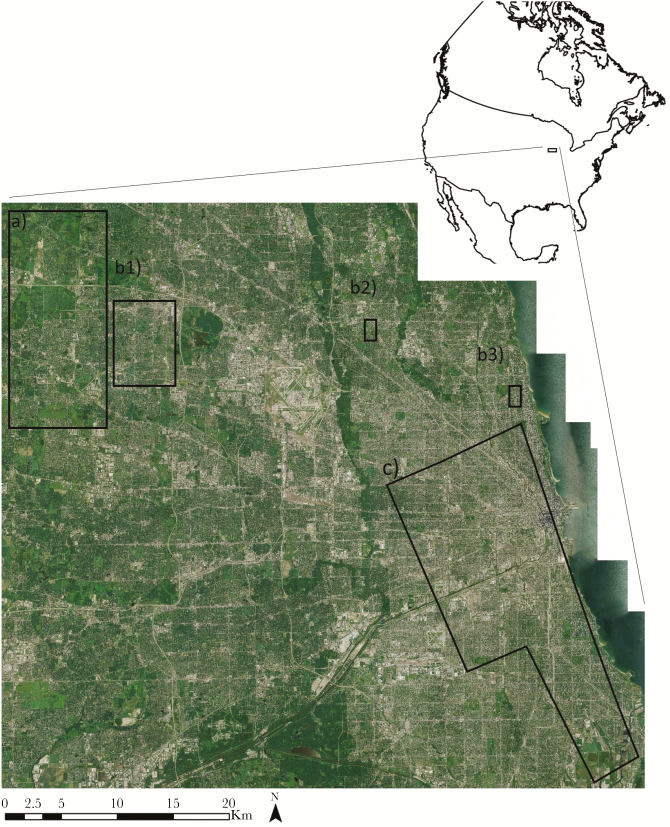
The Chicago metropolitan area, USA, where we monitored resident coyote (*Canis latrans*) space use and movement from 2008 to 2017 in varying levels of urbanization. Natural fragments (a) were dominated by natural land cover such as forest and grassland (often part of the Cook County Forest Preserve system) and were characterized as areas with <20% imperviousness on average. These were surrounded by a low to moderate density of residential land. Suburban landscapes (b1–3) were dominated by a low to moderate density of residential land interspersed with seminatural land cover (e.g., cemeteries and golf courses) and were characterized as areas with an average of 20–50% imperviousness. Highly urbanized landscapes (c) were dominated by high-density residential, commercial, and industrial land with little natural or seminatural land cover and were characterized as an average of >50% imperviousness.

The quality, structure, and spatial dispersion of habitat patches should influence how an animal moves within and among patches (Pyke 1984). Foraging theory predicts that as patch quality increases, the time an animal spends foraging in that patch will increase and, thus, time spent traveling between patches will decrease (MacArthur and Pianka 1966). We might further expect that as food or prey resources within a patch become more predictable or accessible, foraging speed (i.e., the movement speed during a bout of foraging) will increase because searching and handling time will decrease (Schoener 1971). Perceived risk, as a component of patch quality, tends to have a negative relationship with time spent foraging in a patch and a positive relationship with time spent traveling between patches (Brown et al. 1999). At the landscape scale, we might also expect that as perceived risk increases, the time spent encamped (i.e., avoiding perceived risk) will increase. We expect that at the landscape scale, home range size will increase as habitat quality (indexed by a combination of food availability, predictability, accessibility, and perceived risk) decreases (Gittleman and Harvey 1982). Concurrently, as patch dispersion, and thus home range size, increases, we expect that traveling speed between patches will also increase. From these concepts, we propose that trends in movement behavior and animal space use metrics can be used to characterize the behavioral response of wildlife to an urbanization gradient (Table 1) and we use coyotes (*Canis latrans*) occurring across the urbanization gradient in the Chicago metropolitan area, USA, as our model (Figure 1).

**Table 1:** Framework of wildlife responses to urbanization gradients based on movement behavior and space use

	Response to increasing urbanization	
Space use or movement behavior	Positive	Negative
Ratio of time spent foraging to traveling	Increase; individuals travel to fewer or closer patches to meet energetic demands	Decrease; individuals travel to more or farther away patches to meet energetic demands
Foraging speed	Increase; individuals switch to easier and more predictable anthropogenic food items	Stable; individuals have not switched to easier and more predictable anthropogenic food items
Time spent encamped	Stable; individuals do not avoid human presence	Increase; individuals avoid human presence
Home range size	Decrease; less area is needed to meet energetic demands	Increase; more area is needed to meet energetic demands
Home range complexity	Stable, then increase; useable space fragments with large impermeable features, characteristic of highly urbanized landscapes	Increase; useable space fragments with urban features regardless of permeability

Coyotes, widely regarded as highly adaptable, have rapidly colonized most of North America (Gompper 2002; Laliberte and Ripple 2004). The oft-cited reasons for this rapid and near complete colonization are anthropogenic changes to the landscape and to species assemblages (e.g., extirpation of wolves [*Canis lupus*]; Gompper 2002). Indeed, even though the body size of coyotes is smaller than that of wolves, given their body size, life-history traits, and relative trophic position, they are often considered apex predators (Crooks and Soule 1999; Wallach et al. 2015). Coyote populations are known to occur in major urban areas across North America (Gehrt et al. 2010; Poessel et al. 2017). Over the last several decades, we have learned a considerable amount about coyote behavior in urban landscapes. Yet, we know little of how coyotes move within highly urbanized landscapes, as most urban coyote studies have focused on the suburban areas or the periphery of urban areas (e.g., Grinder and Krausman 2001; Gehrt et al. 2009; Poessel et al. 2016). Furthermore, this body of work has produced mixed conclusions about behavioral responses by coyotes to urbanization (summarized by Gehrt et al. 2011). This could be because earlier work often focused only on a few metrics of behavior or on a small segment of the urbanization gradient. Gehrt et al. (2011) found that the demographic response (population density and juvenile survival) of coyotes to urbanization was synanthropic, whereas the space use (home range size), resource selection, activity budget, and dietary responses were indicative of misanthropy or a mixture of synanthropy and misanthropy. More recent work has also produced mixed results. Newsome et al. (2015), for example, found that coyotes consumed more anthropogenically sourced food as urbanization increased, and at the continental scale, Ellington and Murray (2015) found that home range size decreased as the proportion of the landscape with moderate human population density (30–800 people/km^2^) increased—both are examples of positive behavioral responses to urbanization. Other studies, however, have suggested coyotes respond negatively to urbanization. For example, in urban areas, coyotes shifted behavior both spatially and temporally to avoid high human activity or human-dominated land cover types (Mitchell et al. 2015; Poessel et al. 2016).

To better understand the spatial and behavioral responses of an apex predator to urbanization, we considered urbanization across a gradient. We expected that the response of coyotes to urbanization would be nonlinear relative to the degree of urbanization on the landscape. Specifically, we expected coyotes to respond positively to low levels of urbanization but as the degree of urbanization increased, we predicted coyotes would respond negatively (Table 1).

## METHODS

### Study area

The Chicago metropolitan area (CMA) has a human population of over 9 million and is one of the largest urban centers in North America. In 1997, the general land cover across the 6 counties within the CMA was 33% agriculture, 30% urban, 16% natural area, and 21% unassociated vegetation (abandoned agricultural fields and degraded natural lands; Wang and Moskovits 2001). The CMA remains a highly dynamic landscape with agriculture and natural areas being transformed into urban and unassociated vegetation land cover. For the purposes of this analysis, we defined 3 types of urban landscape within the CMA based on the NLCD developed imperviousness data set (Xian et al. 2011; an estimate of the proportion of the surface covered by impenetrable materials, such as asphalt, at a 30-m grid resolution, hereafter called imperviousness). We characterized natural fragments within the urban landscape as areas where the average imperviousness was less than 20%. We describe natural fragments as mostly natural landscape surrounded by low- and medium-density residential and commercial development. These natural fragments were typically part of the Cook County Forest Preserve system. Although these natural areas have minimum development, they are used for recreational activities such as hiking and cross-country skiing, and as such have a year-round human presence at least during daylight (Figure 1). Second, we characterized suburban landscapes as areas where the average imperviousness was between 20% and 50%. We describe suburban landscapes as mostly low- and medium-density residential and commercial development interspersed with small parcels of seminatural land cover (e.g., small parks, cemeteries, golf courses, and water treatment plants; Figure 1). Third, we characterized highly urbanized landscapes as areas where the average imperviousness was greater than 50%. We described highly urbanized landscapes as mainly high-density residential and commercial development or industrialization. Seminatural land cover in highly urbanized landscapes was rare and mainly associated with linear strips along railroad tracks, junkyards, water treatment plants, or landfills except for small lakeshore city parks (Figure 1).

### Animal capture

We trapped coyotes in the Chicago area from April 2008 to January 2016 using leg-hold or snare traps. Once captured, we immobilized animals with Telazol and transported them to a field lab where we collected physical metrics, blood samples, and outfitted them with GPS collars (Lotek WildCell 2008–present, ATS 2013–present). We released animals at capture sites after they recovered from tranquilization. We programed GPS collars to drop after 9–12 months, depending on fix rate schedule and battery life. The typical fix rate was one location every 7.25 h. We further programmed collars to occasionally increase the fix rate to every 3, 2, 1, and 0.25 h, dependent on battery and long-term study objectives. We removed erroneous fix locations by investigating all locations that resulted in a movement speed greater than 10 km/h. If the locations led to impossible movement speeds (>50 km/h) or improbable movements based on individual’s home range and movement patterns, we removed the problematic location. Further details on animal capture and processing can be found in Gehrt et al. (2009). All procedures were approved by Ohio State University’s Institutional Animal Care and Use Committee (Protocol Nos. 2006A0245, 2010A00000113, 2013A00000012), and we followed guidelines of the American Society of Mammalogists (Sikes and Gannon 2011).

### Identifying animal space use strategies

Our GPS location data were collected as part of an extensive, long-term ecological study on urban coyotes; thus, we also had additional information about many collared coyotes (typically 50–70 coyotes monitored each year), such as familial relationships, social status, reproductive status, and space use patterns of mates or other related individuals (via VHF collars). Using a combination of this additional information and observations of space use patterns from the GPS location data, we assigned each individual to one or more space use strategies. We defined residents as animals that maintained a territory, either as an active breeder or as a member of a group of inter-related individuals that also included a breeding pair. We defined transients as animals that used an area that overlapped one or more known resident coyote territories or animals that rarely returned to previously used sites during the monitoring period. To accurately assign an individual to a space use strategy, we required a temporal period of locations > 90 days. Over the monitoring period, some individuals switched space use strategies (Supplementary Appendix 1). We used data from resident coyotes only in subsequent analyses.

### Space use and complexity

To measure space use, we calculated home range size of resident coyotes using the adaptive local convex hull (LoCoH) method (Getz et al. 2007); we used the isopleth nearest to 95% to estimate the home range and the isopleth nearest to 50% to estimate the core home range. We generated LoCoH home ranges using the adaptive method and estimated the adaptive sphere of influence (“a”) as the maximum distance between any 2 locations in the data set (Getz et al. 2007). We also generated home ranges and core areas using 95% and 50% minimum convex polygons (MCP), respectively. Coyote home ranges tend to remain spatially static across seasons (Grinder and Krausman 2001; Gehrt et al. 2009). Thus, variation among animals in the number of locations and length of time observed tends not to influence estimates of home range size to a point; however, less than 90 days of monitoring could result in underestimating home range size. In our study, we used 110–424 days of location data per animal to estimate home ranges. To estimate home range complexity, we capitalized on the differences between the MCP (Mohr 1947; a measure of overall space use) and LoCoH (a measure of useable space) methods to generate a home range complexity index by calculating the difference between LoCoH and MCP isopleths relative to the MCP isopleth ([MCP − LoCoH]/MCP). This index varied from 0 (no complexity, LoCoH and MCP isopleths were identical) to <1 as the difference between LoCoH and MCP increased. We also reported the number of noncontiguous polygons within each LoCoH isopleth. We generated all home range isopleths using the adehabitatHR package (Calenge 2006) in program R (R Core Team 2018) from GPS data that we subsampled to one location approximately every 7.25 h. In this analysis, our sample unit was the home range used. To avoid nonindependence, when we detected home ranges with a high degree of spatial overlap (>90% of space used) and some temporal overlap (thus suggesting that the 2 individuals were part of the same social group), we retained the home range with the greater temporal coverage (Supplementary Appendix 1). Finally, we assigned each unique individual-space use strategy combination to an urban landscape type based on average imperviousness (natural fragment < 20%, suburban 20–50%, highly urbanized > 50%; Xian et al. 2011) within the home range (95% local convex hull).

### Movement behavior

To estimate coyote movement behavior, we subset the GPS location data of resident coyotes such that we retained at least 24 h of consecutive 15-min fixes. Using these criteria, our sample included 51 bursts of GPS location data for 13 individuals, ranging from 24 to 168 h in length. Each burst represents a continuous movement path where each fix is one step in the movement path (Supplementary Appendix 2). We then partitioned the data set into bursts for animals living in each type of urban landscape (natural fragment, suburban, and highly urbanized), assuming that within a landscape type all individuals shared the same movement model. We used the bivariate time series of step lengths and turning angles in this data set to generate movement models based on 2, 3, and 4 movement behavior states with Hidden Markov models (HMM) using the R package moveHMM (Michelot et al. 2016) for each landscape type. We modeled step lengths using a gamma distribution and modeled turning angles using a von Mises distribution, and we added zero inflation to step-length distributions of our models to allow for steps of length zero. Based on the plausible biological interpretation of 2, 3, and 4 movement behavior states, we generated a series of potential step lengths and turning angles to use as starting points for the HMM analysis and ran every combination of these values to ensure that we found the global maximum of the likelihood function (Michelot et al. 2016). We generated predicted movement behavior states for each step (location) using the best models for each number of movement behavior states and urban landscape type using the Viterbi algorithm to decode the underlying unobserved Markov chain (Michelot et al. 2016). Finally, for each step (location), we generated the probability of the predicted movement behavior state given the movement model.

We assumed the 2-state movement model would delineate 2 movement behaviors: encamped, characterized by short step lengths and high turning angles, and moving, characterized by long step lengths and low turning angles. We assumed that the 3-state movement model would delineate encamped behavior and further demarcate moving behavior into foraging, characterized by intermediate step lengths and high turning angles, and traveling, characterized by long step lengths and low turning angles. Finally, we assumed that the 4-state movement model would identify the encamped and traveling movement behaviors, and further distinguish searching behavior, characterized by intermediate step lengths and low turning angles, from foraging behavior, characterized by intermediate step lengths and high turning angles. We assessed model fit and compared the 2-, 3-, and 4-state movement models using 3 criteria:

1) Is the model biologically plausible—here we examined the movement parameters of each movement state to determine whether differences in movement behaviors were biologically realistic.
2) Does the model strongly predict individual movement states for each step—here we used the average likelihood of the most likely movement state at each movement step as an index of predictive power of the movement model and considered a model strongly predictive if this value was ≥0.90.
3) How do biologically plausible and strongly predictive models compare with each other—here we used Akaike information criterion (AIC) to compare models.

## RESULTS

**Figure 2:**
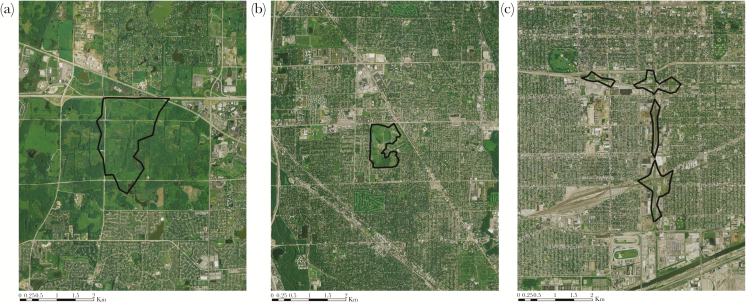
Examples of individual coyote (*Canis latrans*) home ranges estimated using 95% local convex hull polygons in (a) natural fragments, (b) suburban, and (c) highly urbanized landscapes, demonstrating the various sizes and complexity of coyote space use across the urbanization gradient. Coyotes were monitored in the Chicago metropolitan area, USA, from 2008 to 2017.

**Figure 3:**
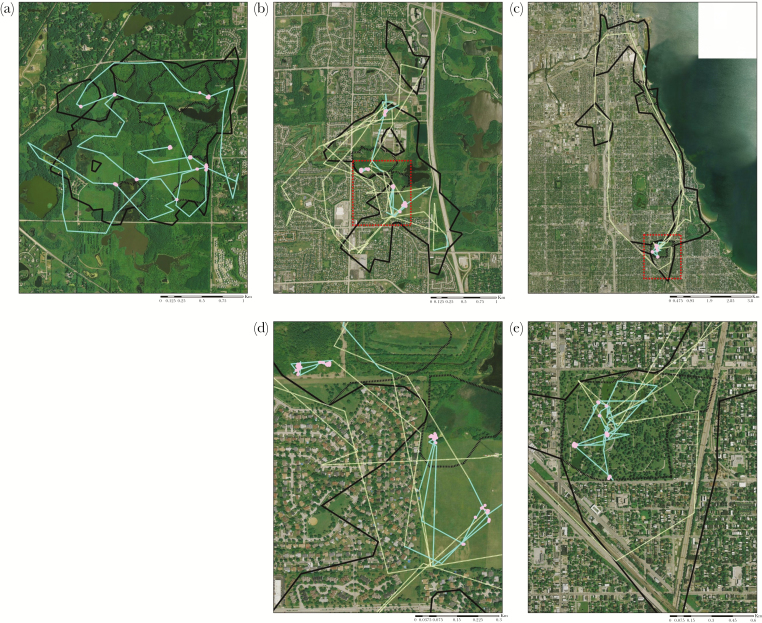
Examples of 2-day windows of movement behavior for 3 coyotes (*Canis latrans*) in the Chicago metropolitan area, USA, from 2008 to 2017. We distinguished only 2 movement behaviors, encamped (pink dots) and moving (blue lines), for coyotes living in natural fragments in the urban landscape (a). For coyotes living in suburban (b) and highly urbanized landscapes (c), we were able to distinguish 3 movement behaviors: encamped (pink dots and lines), foraging (blue lines), and traveling (dashed yellow lines). (d) A zoomed in map of (b), and (e) a zoomed in map of (c), indicated by red squares. Coyote home ranges (95% local convex hull; solid black line) and core ranges (50% local convex hull; small dashed black line) are included for reference.

We captured and monitored 35 coyotes for >90 days, which was long enough to identify space use strategy. From these 35 animals, we identified 23 unique resident coyote home ranges: 7 home ranges in natural fragments, 7 home ranges the suburban landscape, and 9 home ranges in the highly urbanized landscape (Table 2, Figure 2, Supplementary Appendix 1). Furthermore, we identified 51 bursts of continuous 15-min fixes at least 24 h long for 13 coyotes: 13 bursts (4 coyotes) in natural fragments, 13 bursts (3 coyotes) in the suburban landscape, and 25 bursts (6 coyotes) in the highly urbanized landscape. For coyotes in natural fragments, only the 2-state movement model (encamped and moving) produced biologically realistic results, whereas for coyotes in suburban and highly urbanized landscapes, both the 2-state (encamped and moving) and the 3-state (encamped, foraging, traveling) movement models produced biologically realistic and strongly predictive results (Table 3; examples of 2-day windows of movement behavior shown in Figure 3; Supplementary Appendix 3). The 3-state movement model had more support than the 2-state movement model for both suburban (2-state AIC: −2547, 3-state AIC: −3291) and highly urbanized landscapes (2-state AIC: −5478, 3-state AIC: −6746). The 4-state movement model did not produce biologically realistic results, so we did not consider it further.

**Table 2:** Home range and core area size and complexity of coyotes (*Canis latrans*) across 3 urban landscapes within the Chicago metropolitan area, USA, from 2008 to 2017 based on GPS location data collected every 7.5 h

			Complexity^b^		
Urban landscape	Home range^a^ km^2^ (SE)	Core area^a^ km^2^ (SE)	Home range (SE)	Core area (SE)	Noncontiguous polygons^c^ (range)
Natural fragment (*n* = 7)	3.24 (0.32)	0.65 (0.08)	0.20 (0.03)	0.35 (0.03)	1.29 (1–2)
Suburban (*n* = 7)	2.31 (1.11)	0.22 (0.10)	0.43 (0.06)	0.16 (0.03)	2.14 (1–5)
Highly urbanized (*n* = 9)	7.05 (2.42)	0.78 (0.31)	0.65 (0.07)	0.30 (0.03)	1.89 (1–4)

^a^We calculated core area (50% isopleth) and home range (95% isopleth) with the LoCoH in the adehabitatHR package (Calenge 2006) in R (R Core Team 2018).

^b^We calculated home range and core area complexity index using the formula: (MCP − LoCoH)/MCP, where MCP is the 95% (50%) MCP.

^c^Noncontiguous polygons represent an additional index of home range complexity.

As urbanization increased from low to moderate levels (i.e., from natural fragments to the suburban landscape), we found both positive and negative responses to increased urbanization by coyotes. Average home range size (95% LoCoH) decreased from 3.24 km^2^ (SE = 0.32) to 2.31 km^2^ (SE = 1.11; Table 2). This trend was consistent with a positive response to urbanization, suggesting that the landscape quality perceived by coyotes was higher in suburban areas (20–50% imperviousness) than natural fragments (<20% imperviousness). Conversely, the average home range complexity index significantly increased from 0.20 (SE = 0.03) in the natural fragments to 0.43 (SE = 0.06; *P* ≤ 0.01; Table 2) in the suburban landscape, suggesting that coyotes viewed suburban landscapes as more fragmented, consistent with a negative response to urbanization. We were unable to distinguish foraging and traveling behaviors of coyote in natural fragments (only the 2-state model produced biologically realistic results); this precluded a comparison of relative foraging time to traveling time as urbanization increased from low to moderate. However, the predicted proportion of time spent encamped (as defined by the 2-state movement model) was similar between natural fragments (0.57) and suburban landscapes (0.58). This suggests that coyotes were not responding to increased human presence in suburban landscapes by spending more time encamped.

As urbanization increased from moderate to high (i.e., from suburban to highly urbanized landscapes), we found that coyotes mostly had negative behavioral responses. The average home range size increased from 1.25 km^2^ (SE = 0.40) in the suburban landscape to 7.05 km^2^ (SE = 0.65) in the highly urbanized landscape (*P* = 0.02; Table 2). The home range complexity index also increased from 0.43 (SE = 0.06) in the suburban landscape to 0.65 (SE = 0.07) in the highly urbanized landscape (*P* = 0.02; Table 2). In highly urbanized landscapes, coyotes spent slightly more time encamped (0.58 in the suburban landscape and 0.62 in the highly urbanized landscape; Table 3), suggesting that coyotes are attempting to avoid the increased human presence. The distribution of foraging step length did not appear to change as urbanization increased (suburban: average = 130 m [SE = 114] and highly urbanized: average = 122 m [SE = 114]; Table 3; Supplementary Appendix 3). This suggests that the increase in urbanization did not induce a change in coyote foraging behavior. Interestingly, the foraging to traveling ratio of 1.34 (Table 3) was driven by one individual (coyote 434) in the suburban landscape who spent very little time traveling and had a foraging to traveling ratio of 7.20 (Supplementary Appendix 4). When we removed this individual from the analysis, the foraging to traveling ratio in the suburban landscape was 0.81, a ratio that is lower than that of the highly urbanized landscape (1.06; Table 3). This suggests that coyotes in highly urbanized landscapes spend more time foraging and less time traveling than coyotes in suburban landscapes. We interpret this as a positive response to urbanization because individuals travel to fewer or closer patches to meet energetic demands.

**Table 3:** Movement characteristics and behavior of coyotes (*Canis latrans*) across 3 urban landscapes within the Chicago metropolitan area, USA, from 2008 to 2017 based on GPS location data collected every 15 min

			2-state movement model			3-state movement model^b^				
			Encamped		Moving	Foraging		Traveling		Time spent foraging:traveling
Urban landscape	*n* ^a^	Number of 15-min segments	Step length in meters (SD) [minimum, maximum]	Time spent (prop)	Step length in meters (SD) [minimum, maximum]	Step length in meters (SD) [minimum, maximum]	Time spent (prop)	Step length in meters (SD) [minimum, maximum]	Time spent (prop)	
Natural fragment	3	2311	7.4 (8.3) [0–57]	0.57	306 (238) [3–1424]	—	—	—	—	—
Suburban	3	5210	7.6 (7.2) [0–54]	0.58	319 (256) [0.2–1287]	130 (114) [0.2–729]	0.25	528 (218) [108–1287]	0.19	1.34
Highly urbanized	6	7909	7.5 (7.2) [0–52]	0.62	390 (378) [0–2985]	122 (114) [0–830]	0.21	625 (385) [44–2985]	0.20	1.06

^a^Number of individuals.

^b^The 3-state movement model did not produce biologically plausible results for coyotes in natural fragments. Given our data set and movement characteristics, foraging and traveling were indistinguishable for coyotes in natural fragments.

Individual variability in space use patterns increased as urbanization increased and was greatest in highly urbanized landscapes. For example, home range size in highly urbanized landscapes ranged from 1.26 to 23.12 km^2^ and complexity ranged from 0.18 to 0.89 (Supplementary Appendix 4). This wide range appears to be driven by 3 individuals that occupied very large home ranges that spanned long swaths of the Lake Michigan shoreline immediately adjacent to the city center of Chicago (coyotes 441, 855, 866; Supplementary Appendix 5). In addition, coyote 885 occupied a home range in the highly urbanized landscape that had relatively low complexity—this home range was centered on a landfill and recycling plant along the heavily industrialized Calumet River (Supplementary Appendix 5). The home range of one individual (coyote 298) that occupied the suburban landscape was larger than other individuals monitored in the suburban landscape (Supplementary Appendix 5). These outliers influenced the average home range size and complexity estimates, but not sufficient to change the interpretation of the response of home range size and complexity to urbanization.

## DISCUSSION

In highly urbanized landscapes, coyotes demonstrated mostly negative responses to urbanization. Relative to suburban landscapes, coyotes viewed highly urbanized landscapes as lower quality (increased home range size), more fragmented (increased home range complexity), and riskier (increased time spent encamped), and their foraging speed was unchanged. However, coyotes appeared to view highly urbanized landscapes as having higher quality patches or patches that were closer (decreased traveling relative to foraging) relative to suburban landscapes. Conversely, relative to natural fragments, coyotes viewed the suburban landscape as higher quality (decreased home range size) and equally risky (time spent encamped unchanged), but more fragmented (increased home range complexity). Our results suggest that coyotes can respond positively to moderate levels of urbanization and can persist in highly urbanized landscapes, yet their behavior is not entirely consistent with what we would expect from a synanthropic species.

Other studies in the CMA have found that in suburban areas, coyotes incorporate more anthropogenic food into their diet while at the same time avoid most areas associated with humans (Gehrt et al. 2009, 2011; Newsome et al. 2015). This utilization of anthropogenic food sources could increase the quality of suburban landscapes for coyotes, allowing them to persist in smaller home ranges. At the same time, by spatially avoiding humans, coyotes do not need to spend more time encamped. There is, however, some disagreement among our study and earlier studies in the same system. For example, Gehrt et al. (2009, 2011) found that home range size increased as urbanization increased from low to moderate levels, whereas we found that home range size decreased. These differences could be attributed to how we measured home range size: we used a method (LoCoH) that is less susceptible to inflating home range size when home ranges are complex (i.e., highly linear or noncontiguous). In addition, Gehrt et al. (2009, 2011) used VHF telemetry data and not GPS telemetry data (the 95% MCP is thought to be less susceptible to underrepresenting home range size when using VHF telemetry data). Moreover, these studies occurred in mutually exclusive time periods, and although our study area overlapped spatially with that of Gehrt et al. (2009, 2011), our study area encompassed a larger urbanization gradient.

There is little data reported in the literature about how carnivores respond to highly urbanized landscapes. Globally, there are several apex carnivore species known to occupy landscapes with low to moderate urbanization (e.g., Gehrt et al. 2009; Bateman and Fleming 2012; Smith et al. 2015), but to our knowledge, this is the first study to describe space use and movement of coyotes or any other apex carnivore within highly urbanized landscapes (defined as >50% imperviousness). Seemingly, the delicate balance coyotes maintained in suburban landscapes breaks down in highly urbanized landscapes, where coyotes spend more time encamped, presumably because they cannot spatially avoid humans and because they view the landscape as highly fragmented. In highly urbanized landscapes, the balance between natural and anthropogenic food sources probably tips further in favor of anthropogenic sources, yet we found that coyotes either do not increase their reliance on anthropogenic food sources or they do, but this increased reliance does not result in a detectable increase in our estimate of coyote foraging speed. Perhaps in highly urbanized landscapes both anthropogenic and natural food sources are scarcer but more clumped than in suburban landscapes—this could explain the lack of response in coyote foraging speed and could be a driver of the increase in the ratio of foraging to traveling. Yet, despite the limitations of highly urbanized landscapes, coyotes can persist in these landscapes. Indeed, highly urbanized landscapes are unlikely to be the most resource-limited landscapes coyotes inhabit—coyotes persist in boreal forests and the barrens of Gaspé Peninsula and Newfoundland, Canada, with some of the largest home ranges reported (Boisjoly et al. 2009; Ellington 2015), presumably because food resources are scarce.

In highly urbanized landscapes, however, not only do coyotes appear to experience lower prey availability, our findings suggest that they also view these landscapes as risky and highly fragmented. Smith et al. (2015, 2017) found that cougars (*Puma concolor*) reduced feeding time in response to perceived human presence and linked this behavioral shift to increased predation rates (if not consumption rates) by cougars in urbanized environments. If coyotes respond to perceived human risk in highly urbanized environments in a similar way (i.e., increased predation rates but reduced feeding time at individual foraging events), then coyotes could exhibit increased predation on natural prey, increased human–wildlife conflict (via increased foraging rates on anthropogenic food), or both. Understanding the link between our findings and diet and resource selection in highly urbanized landscape would be an important step toward understanding the drivers of coyote behavior in these landscapes.

Our inability to differentiate foraging and traveling behavior in natural areas could be driven by relatively low sample size (the sample size of movement paths in natural fragments was smaller than the other urban landscapes) or it could be biological. If foraging and traveling behavior are biologically indistinguishable, it could mean that coyotes are always foraging when they are moving, indicating that patch dispersion in natural areas is such that there is little need to exclusively travel between patches. We might expect this behavior to arise in home ranges with little fragmentation and uniform prey availability. Conversely, foraging and traveling behavior could still be distinct in natural fragments, but our sampling rate was insufficient to capture the scale at which these behaviors were distinct.

A prominent feature of ecological research on coyotes is the high degree of individual variation observed in ecological responses (Gehrt et al. 2009; Murray and St. Clair 2015; Newsome et al. 2015). We found high individual variation within urban landscapes for every metric we measured. Undoubtedly, some of this variation was driven by the variation in landscape occupied by individual coyotes within each urban landscape class. For example, the variability in home range size and complexity in the highly urbanized landscape might be linked to very different types of features on this urbanized landscape. Murray and St. Clair (2017) found that even microsite differences influenced the likelihood of residential yards being used by coyotes. Newsome et al. (2015) found that individual variation in the use of anthropogenically sourced food was stronger than any population-level response by coyotes to urbanization. Individual variation in response to urbanization can also be the result of genetic differences (Partecke et al. 2006), personality differences (Scales et al. 2011), or social environment differences (Gehrt et al. 2009); exploring the relationship between these factors and urbanization will be an interesting avenue of future research.

Like many ecological studies, there could be confounding factors in our analysis. For example, average home range size on a given landscape can change over time due to temporal variation in population density and increased competition (Lopez-Sepulcre and Kokko 2005). It is unknown whether the coyotes in our study encountered the same level of competitive pressure by conspecifics, driven by population density, in each type of urban landscape. Second, our categorization of movement behavior into 3 types (encamped, foraging, and traveling) is relatively simplistic. In addition to foraging, moving coyotes also mark and defend territories, interact socially with conspecifics, and avoid predators (humans). Our study design would probably have classified these behaviors as foraging. Furthermore, long stationary foraging bouts (e.g., consuming an adult white-tailed deer [*Odocoileus virginianus*] carcass) could have been classified as encamped behavior. It is also unlikely that the purpose of all behavior that we labeled as traveling was to arrive at a new foraging patch. Instead, we also expect traveling behavior to encompass movement driven by territorial defense and escaping real or perceived risks. Our analysis did not allow us to differentiate these behaviors; thus, we assumed that the proportion of time spent engaging in territorial and social behaviors did not vary between urban landscapes. It is possible that risk-fleeing behavior increased as urbanization increased, and our study design was unable to capture this. We did, however, observe coyotes making other behavioral changes (i.e., increased time spent encamped), in line with risk avoidance. Future studies that directly link diet and resource selection with movement behavior might better elucidate these relationships.

The rate of global biodiversity loss has been on the rise (Johnson et al. 2017), in part, because of urbanization (Czech et al. 2000). As urbanization continues to alter natural environments, it is imperative that we expand our efforts to understand the mechanisms driving space use and behavioral responses to these human-altered landscapes. We used animal movement models and concepts from foraging theory to characterize the behavioral response of an apex predator, coyotes, to an urbanization gradient. Although some responses were linear, urbanization fragments the landscape, and the response of coyotes to urbanization was nonlinear and more complex. At moderate levels of urbanization, coyotes appear to respond positively to urbanization and only begin to respond negatively at high levels of urbanization. Yet, despite these limitations, coyotes, as an apex predator, persist in highly urbanized landscapes, which suggests more complex trophic systems could exist in a highly urbanized landscape than previously thought.

## FUNDING

This work was supported by Cook County Animal and Rabies Control, the Max McGraw Wildlife Foundation, and Forest Preserve District of Cook County.

## 

Authors’ contribution: E.H.E. and S.D.G. conceived the ideas and designed the methodology. S.D.G. collected the data. E.H.E. analyzed the data and led the writing of the manuscript. Both authors contributed critically to the manuscript drafts and gave final approval for submission.

Conflict of interest: The authors have no competing interests.

Data accessibility: Analyses reported in this article can be reproduced using the data provided by Ellington and Gehrt (2019).

## Supplementary Material

arz019_suppl_Appendix-1-4

arz019_suppl_Map_Appendix
