# Supplementary material for: Behavioral responses by an apex predator to urbanization
Source: Behav Ecol. 2019 Mar 2;30(3):821–9. doi: 10.1093/beheco/arz019 (PMC6562302; doi:10.1093/beheco/arz019)
Supplement: arz019_suppl_Appendix-1-4 [file arz019_suppl_appendix-1-4.docx]

Appendix 1. Summary of coyote (*Canis latrans*) space use in the Chicago Metropolitan Area

Table 1. Space use of individual coyotes in the Chicago Metropolitan Area. Locations were collected using GPS collars from 2008-2017

| ID | Urban landscape | Average percent of developed imperviousness | First location (mm/dd/yyyy hh:mm) | Last location (mm/dd/yyyy hh:mm) | Days | Core area (km^2^)^a^ | Home range (km^2^)^a^ | 50% MCP (km^2^) | 95% MCP (km^2^) | Home range complexity^b^ | Core area complexity^b^ |
| --- | --- | --- | --- | --- | --- | --- | --- | --- | --- | --- | --- |
| 164 | Natural | 4.4 | 04/11/2013 21:45 | 09/16/2013 05:01 | 157.30 | 0.63 | 2.90 | 0.83 | 3.20 | 0.09 | 0.26 |
| 227 | Suburban | 31.5 | 05/23/2008 01:00 | 04/02/2009 02:16 | 314.05 | 0.30 | 2.90 | 0.49 | 6.59 | 0.56 | 0.07 |
| 298 | Suburban | 40.4 | 06/12/2008 19:02 | 03/10/2009 09:16 | 270.59 | 0.77 | 8.67 | 2.98 | 11.30 | 0.23 | 0.26 |
| 308 | Suburban | 23.2 | 05/18/2009 02:15 | 09/08/2009 19:02 | 113.70 | 0.23 | 1.75 | 0.40 | 4.60 | 0.62 | 0.09 |
| 321 | Suburban | 29.9 | 04/28/2008 16:45 | 06/26/2009 07:03 | 423.60 | 0.09 | 0.72 | 0.21 | 1.01 | 0.29 | 0.21 |
| 362 | Natural | 2 | 05/17/2014 00:30 | 03/09/2015 20:31 | 296.83 | 0.46 | 2.22 | 1.08 | 3.43 | 0.35 | 0.31 |
| 370 | Natural | 1.3 | 04/16/2009 09:32 | 04/16/2010 02:15 | 364.70 | 0.69 | 3.23 | 1.56 | 4.05 | 0.20 | 0.38 |
| 434 | Suburban | 25.9 | 02/19/2010 18:00 | 06/29/2010 02:15 | 129.30 | 0.01 | 0.27 | 0.05 | 0.51 | 0.46 | 0.09 |
| 441 | Highly Urbanized | 62.2 | 03/10/2010 22:02 | 07/27/2010 23:02 | 139.00 | 3.13 | 23.12 | 18.07 | 54.79 | 0.58 | 0.33 |
| 448 | Natural | 2.7 | 12/18/2010 08:00 | 08/27/2011 11:15 | 252.09 | 0.53 | 3.15 | 1.54 | 3.79 | 0.17 | 0.41 |
| 672 | Natural | 3 | 01/09/2013 20:47 | 09/04/2013 11:00 | 237.55 | 0.53 | 2.61 | 1.14 | 3.02 | 0.14 | 0.38 |
| 678 | Natural | 3.8 | 04/10/2015 22:45 | 10/08/2015 15:30 | 180.70 | 0.65 | 3.86 | 2.60 | 5.26 | 0.27 | 0.49 |
| 695 | Natural | 16.3 | 10/31/2015 22:45 | 05/31/2016 09:45 | 212.46 | 1.09 | 4.73 | 1.40 | 5.65 | 0.16 | 0.25 |
| 740 | Suburban | 23.5 | 06/26/2013 19:32 | 03/12/2014 15:00 | 258.81 | 0.04 | 0.46 | 0.12 | 0.68 | 0.32 | 0.18 |
| 744 | Highly Urbanized | 72.1 | 11/13/2013 06:01 | 11/24/2014 04:16 | 375.93 | 0.75 | 3.76 | 2.72 | 8.47 | 0.56 | 0.32 |
| 748 | Highly Urbanized | 60.6 | 02/24/2014 21:00 | 06/14/2014 15:46 | 109.74 | 0.33 | 3.41 | 5.25 | 14.28 | 0.76 | 0.37 |
| 750 | Highly Urbanized | 78.3 | 03/20/2014 05:23 | 11/25/2014 23:45 | 250.81 | 0.68 | 5.24 | 3.04 | 16.70 | 0.69 | 0.18 |
| 854 | Suburban | 27.5 | 01/27/2016 23:00 | 02/24/2017 06:45 | 383.24 | 0.12 | 1.38 | 0.59 | 2.80 | 0.51 | 0.21 |
| 855 | Highly Urbanized | 59.1 | 11/22/2014 23:01 | 09/18/2015 11:30 | 299.48 | 0.95 | 13.42 | 31.24 | 52.03 | 0.74 | 0.60 |
| 866 | Highly Urbanized | 53.5 | 01/14/2015 06:15 | 09/22/2015 08:01 | 251.03 | 0.35 | 9.56 | 4.51 | 27.61 | 0.65 | 0.16 |
| 885 | Highly Urbanized | 65.2 | 05/06/2015 03:30 | 03/05/2016 07:16 | 304.20 | 0.38 | 2.08 | 0.90 | 2.53 | 0.18 | 0.36 |
| 970 | Highly Urbanized | 78.2 | 12/17/2015 22:01 | 12/15/2016 16:00 | 363.75 | 0.16 | 1.26 | 2.11 | 7.37 | 0.83 | 0.29 |
| 971 | Highly Urbanized | 79.5 | 01/10/2016 06:00 | 11/16/2016 03:45 | 310.91 | 0.24 | 1.61 | 1.51 | 14.88 | 0.89 | 0.10 |
| ***Individuals excluded from the analysis*** | | | | | | | | | | | |
| 352^c^ | Suburban | 27.7 | 12/17/2008 06:00 | 05/04/2009 11:00 | 138.17 | 0.09 | 1.17 | 0.17 | 1.87 | 0.38 | 0.09 |
| 446^c^ | Natural | 5.9 | 05/06/2011 20:30 | 12/24/2011 03:30 | 231.33 | 0.30 | 2.31 | 0.65 | 3.33 | 0.31 | 0.20 |
| 516^c^ | Natural | 4.2 | 04/17/2013 21:46 | 08/16/2013 17:01 | 120.80 | 0.70 | 2.90 | 1.10 | 3.34 | 0.13 | 0.33 |
| 972^c^ | Highly Urbanized | 79.6 | 12/19/2015 14:30 | 07/27/2016 09:15 | 220.74 | 0.13 | 2.96 | 0.84 | 10.92 | 0.73 | 0.08 |
| 800^d^ | Natural | 13.3 | 03/09/2016 18:00 | 12/16/2016 14:30 | 281.85 | 0.17 | 1.10 | 0.69 | 1.83 | 0.40 | 0.38 |
| 434^e^ | Suburban | 38.1 | 06/29/2010 09:31 | 11/05/2010 11:00 | 129.06 | 0.65 | 7.77 | 4.36 | 27.99 | 0.72 | 0.16 |
| 584^e^ | Suburban |  | 01/10/2013 18:31 | 07/04/2013 03:01 | 174.31 |  |  |  |  |  |  |
| 695^e^ | Suburban | 14.4 | 03/19/2015 21:00 | 10/31/2015 15:30 | 225.77 | 1.95 | 15.38 | 4.77 | 31.33 | 0.51 | 0.15 |
| 725^e^ | Natural | 4.9 | 05/26/2014 07:30 | 02/01/2015 13:30 | 251.29 | 1.14 | 8.58 | 4.42 | 17.16 | 0.50 | 0.26 |
| 873^e^ | Suburban | 24.6 | 04/13/2015 22:45 | 03/05/2016 00:00 | 326.09 | 4.13 | 26.82 | 11.61 | 49.00 | 0.45 | 0.24 |
| 880^e^ | Natural | 7.6 | 04/09/2015 08:15 | 05/27/2016 20:30 | 414.51 | 1.40 | 9.24 | 6.87 | 16.23 | 0.43 | 0.42 |
| 882^e^ | Highly Urbanized | 73.4 | 04/09/2015 15:03 | 03/31/2016 08:46 | 356.74 | 3.99 | 31.52 | 26.60 | 93.19 | 0.66 | 0.29 |

^a^ We calculated core area and home range with the local convex hull (LoCoH) isopleth nearest 50% and 95%, respectively, with the adehabitatHR package (Calenge 2006) in R (R Core Team 2017)

^b^ We calculated the complexity index using the formula: (MCP – LoCoH)/MCP, where MCP is the 95% minimum convex polygon

^c^ These individuals shared a resident home range with another animal that was monitored for a longer time

^d^ This individual was excluded from the analysis because the LoCoH estimation did not optimize properly, thus it was an underestimation of space use and overestimation of complexity.

^e^ These individuals were transients that used an area that overlapped one or more known resident coyote territories or rarely returned to previously used sites during the monitoring period.

Appendix 2. Movement characteristics of coyotes (*Canis latrans*) in the Chicago Metropolitan Area.

Table 1. Movement characteristics of individual bursts of continuous coyote steps (15 minutes) in the Chicago Metropolitan Area from 2008 to 2017. We applied a two-state movement model to bursts to identify two movement behaviors, encamped and moving.

| ID | Urban landscape | First location (mm/dd/yyyy hh:mm) | Last location (mm/dd/yyyy hh:mm) | Steps | Encamped | | | Moving | | |
| --- | --- | --- | --- | --- | --- | --- | --- | --- | --- | --- |
|  |  |  |  |  | Time spent  (prop) | Average step-length (m; SD) | Step-length range (m) | Time spent  (prop) | Average step-length (m; SD) | Step-length range (m) |
| 227 | Suburban | 8/21/2008 0:00 | 8/22/2008 23:45 | 192 | 0.59 | 8.1 (7.5) | 0.1 - 40.2 | 0.41 | 375 (269) | 3 - 1031 |
| 434 | Suburban | 2/24/2010 1:00 | 2/28/2010 11:00 | 425 | 0.66 | 5.7 (5.8) | 0 - 39.2 | 0.34 | 152 (94) | 9 - 420 |
| 434 | Suburban | 3/12/2010 1:00 | 3/15/2010 10:45 | 328 | 0.66 | 7 (8.4) | 0.2 - 51.9 | 0.34 | 173 (147) | 5 - 699 |
| 434 | Suburban | 4/14/2010 1:00 | 4/18/2010 11:00 | 425 | 0.54 | 6.4 (6.2) | 0.1 - 35.3 | 0.46 | 200 (160) | 1 - 1015 |
| 434 | Suburban | 5/12/2010 1:00 | 5/14/2010 10:45 | 232 | 0.63 | 7.6 (7.6) | 0 - 42.8 | 0.37 | 217 (182) | 5 - 873 |
| 434 | Suburban | 6/18/2010 2:00 | 6/19/2010 15:15 | 150 | 0.58 | 6.9 (5.4) | 1 - 24.4 | 0.42 | 163 (153) | 3 - 811 |
| 434 | Suburban | 6/20/2010 19:30 | 6/21/2010 20:15 | 100 | 0.70 | 6.1 (5.2) | 0.4 - 27.1 | 0.30 | 107 (91) | 9 - 399 |
| 854 | Suburban | 3/24/2016 0:00 | 3/30/2016 23:45 | 672 | 0.60 | 10 (8.9) | 0 - 54.5 | 0.40 | 381 (240) | 1 - 1030 |
| 854 | Suburban | 5/24/2016 0:00 | 5/25/2016 8:00 | 129 | 0.36 | 11.7 (7.1) | 1 - 30.1 | 0.64 | 331 (265) | 2 - 1083 |
| 854 | Suburban | 5/25/2016 8:30 | 5/30/2016 23:45 | 542 | 0.57 | 8.7 (7.2) | 0.1 - 46 | 0.43 | 362 (277) | 1 - 1099 |
| 854 | Suburban | 7/24/2016 0:00 | 7/30/2016 23:45 | 672 | 0.56 | 8.3 (7.1) | 0.1 - 42.8 | 0.44 | 372 (285) | 0 - 1287 |
| 854 | Suburban | 11/24/2016 0:00 | 11/30/2016 23:45 | 672 | 0.54 | 8.1 (7.6) | 0.2 - 54.1 | 0.46 | 384 (281) | 1 - 1235 |
| 854 | Suburban | 1/24/2017 0:15 | 1/30/2017 23:45 | 671 | 0.61 | 5.6 (5.5) | 0.1 - 42.7 | 0.39 | 400 (251) | 1 - 1100 |
| 370 | Natural | 2/28/2010 0:00 | 3/4/2010 0:00 | 385 | 0.65 | 8.1 (6.3) | 0.6 - 41 | 0.35 | 287 (182) | 15 - 982 |
| 672 | Natural | 5/15/2013 19:45 | 5/18/2013 2:00 | 218 | 0.34 | 15.3 (10.2) | 1.5 - 40.6 | 0.66 | 244 (183) | 17 - 740 |
| 672 | Natural | 5/18/2013 16:45 | 5/21/2013 3:15 | 235 | 0.52 | 13.7 (10.5) | 0.5 - 56.8 | 0.48 | 262 (195) | 14 - 962 |
| 672 | Natural | 5/21/2013 15:00 | 5/22/2013 23:45 | 132 | 0.47 | 10.9 (7.9) | 0.5 - 31.4 | 0.53 | 266 (195) | 19 - 804 |
| 678 | Natural | 5/19/2015 9:00 | 5/20/2015 23:00 | 153 | 0.48 | 5.5 (6.1) | 0 - 29.1 | 0.52 | 266 (263) | 3 - 1300 |
| 678 | Natural | 5/21/2015 9:45 | 5/22/2015 11:15 | 103 | 0.48 | 7.2 (7.2) | 0.8 - 27.7 | 0.52 | 336 (282) | 34 - 1265 |
| 678 | Natural | 5/22/2015 11:45 | 5/25/2015 4:00 | 258 | 0.68 | 5.8 (7.6) | 0 - 44.5 | 0.32 | 334 (239) | 9 - 970 |
| 678 | Natural | 5/29/2015 2:45 | 5/30/2015 10:45 | 129 | 0.50 | 3.3 (3.4) | 0 - 17.9 | 0.50 | 317 (278) | 19 - 1223 |
| 695 | Natural | 5/19/2016 11:45 | 5/21/2016 7:00 | 174 | 0.65 | 4.8 (9.3) | 0 - 45.8 | 0.35 | 392 (303) | 49 - 1424 |
| 695 | Natural | 5/21/2016 7:30 | 5/23/2016 0:30 | 165 | 0.59 | 4.1 (3.5) | 0 - 16.8 | 0.41 | 389 (271) | 9 - 1042 |
| 695 | Natural | 5/24/2016 6:00 | 5/25/2016 19:30 | 151 | 0.58 | 6.9 (9.5) | 0 - 49.3 | 0.42 | 324 (270) | 9 - 1397 |
| 695 | Natural | 5/25/2016 21:30 | 5/27/2016 0:45 | 110 | 0.61 | 6 (8) | 0 - 35.5 | 0.39 | 351 (218) | 54 - 1007 |
| 695 | Natural | 5/27/2016 2:00 | 5/28/2016 2:15 | 98 | 0.68 | 3 (2.8) | 0 - 14.9 | 0.32 | 433 (264) | 43 - 950 |
| 750 | Highly Urbanized | 11/2/2014 20:15 | 11/4/2014 23:45 | 207 | 0.70 | 7.2 (6.7) | 0.4 - 48.3 | 0.30 | 317 (271) | 15 - 1076 |
| 855 | Highly Urbanized | 12/9/2014 0:00 | 12/12/2014 23:45 | 384 | 0.47 | 5.8 (5.5) | 0.1 - 25.4 | 0.53 | 451 (398) | 4 - 1784 |
| 855 | Highly Urbanized | 2/9/2015 1:30 | 2/12/2015 7:00 | 311 | 0.58 | 6.8 (7) | 0 - 43.8 | 0.42 | 511 (437) | 5 - 1618 |
| 855 | Highly Urbanized | 5/9/2015 0:00 | 5/11/2015 1:30 | 199 | 0.60 | 5.7 (5.4) | 0.4 - 27 | 0.40 | 448 (364) | 0 - 1534 |
| 855 | Highly Urbanized | 5/11/2015 2:00 | 5/13/2015 0:00 | 185 | 0.72 | 8.9 (7.8) | 0.1 - 44.2 | 0.28 | 542 (564) | 31 - 2592 |
| 855 | Highly Urbanized | 7/9/2015 0:00 | 7/12/2015 23:45 | 384 | 0.52 | 7.4 (6.9) | 0.3 - 42.9 | 0.48 | 601 (610) | 2 - 2556 |
| 855 | Highly Urbanized | 9/9/2015 0:00 | 9/12/2015 6:45 | 316 | 0.58 | 10.2 (9.2) | 0.5 - 46.8 | 0.42 | 490 (444) | 1 - 1965 |
| 866 | Highly Urbanized | 3/9/2015 0:00 | 3/12/2015 23:45 | 384 | 0.70 | 8.3 (6.6) | 0.4 - 40.2 | 0.30 | 280 (265) | 4 - 1747 |
| 866 | Highly Urbanized | 5/9/2015 1:00 | 5/12/2015 14:45 | 344 | 0.62 | 6.8 (7.1) | 0.1 - 39.8 | 0.38 | 392 (344) | 8 - 1432 |
| 866 | Highly Urbanized | 7/9/2015 0:00 | 7/11/2015 15:45 | 256 | 0.56 | 8.6 (7) | 0.2 - 35.1 | 0.44 | 258 (258) | 7 - 1399 |
| 866 | Highly Urbanized | 7/11/2015 16:15 | 7/12/2015 23:45 | 127 | 0.73 | 8.9 (7.5) | 0.8 - 42.3 | 0.27 | 414 (337) | 8 - 1227 |
| 866 | Highly Urbanized | 9/9/2015 0:00 | 9/12/2015 9:00 | 325 | 0.65 | 8.6 (7.7) | 0 - 46.3 | 0.35 | 408 (364) | 9 - 1455 |
| 885 | Highly Urbanized | 5/9/2015 8:00 | 5/11/2015 9:45 | 200 | 0.45 | 5.2 (4.1) | 0.3 - 22.2 | 0.56 | 475 (335) | 3 - 1428 |
| 885 | Highly Urbanized | 7/9/2015 0:00 | 7/10/2015 14:30 | 155 | 0.60 | 6.3 (5.6) | 0.2 - 29.2 | 0.40 | 454 (356) | 29 - 1396 |
| 885 | Highly Urbanized | 7/10/2015 15:00 | 7/12/2015 20:30 | 215 | 0.66 | 6.6 (4.4) | 0.9 - 23.2 | 0.34 | 419 (345) | 3 - 1308 |
| 885 | Highly Urbanized | 9/10/2015 16:45 | 9/12/2015 1:15 | 131 | 0.71 | 7.3 (8.1) | 0.1 - 38.1 | 0.29 | 444 (319) | 12 - 1238 |
| 970 | Highly Urbanized | 1/24/2016 0:00 | 1/30/2016 23:45 | 672 | 0.67 | 5.9 (5.9) | 0.1 - 37.4 | 0.33 | 373 (312) | 17 - 1401 |
| 970 | Highly Urbanized | 5/24/2016 0:00 | 5/30/2016 19:00 | 653 | 0.61 | 9.8 (9.8) | 0.1 - 52.3 | 0.39 | 278 (277) | 0 - 1537 |
| 970 | Highly Urbanized | 7/25/2016 23:30 | 7/29/2016 17:15 | 360 | 0.61 | 8.9 (8.3) | 0.3 - 46.1 | 0.39 | 233 (227) | 4 - 939 |
| 970 | Highly Urbanized | 7/29/2016 18:45 | 7/30/2016 23:45 | 117 | 0.63 | 9.9 (7.6) | 0.5 - 32.9 | 0.37 | 251 (235) | 15 - 814 |
| 970 | Highly Urbanized | 11/24/2016 0:00 | 11/30/2016 23:45 | 672 | 0.56 | 5.6 (5.1) | 0 - 38 | 0.44 | 339 (293) | 3 - 1399 |
| 971 | Highly Urbanized | 1/24/2016 0:00 | 1/28/2016 9:45 | 424 | 0.57 | 6.2 (6.1) | 0 - 35.9 | 0.43 | 382 (356) | 0 - 1512 |
| 971 | Highly Urbanized | 5/23/2016 23:45 | 5/25/2016 20:45 | 181 | 0.76 | 9.7 (9.3) | 0.5 - 47.7 | 0.24 | 329 (303) | 15 - 1191 |
| 971 | Highly Urbanized | 5/27/2016 12:00 | 5/30/2016 23:45 | 336 | 0.75 | 8.7 (7.2) | 0.5 - 38.5 | 0.25 | 329 (312) | 2 - 1596 |
| 971 | Highly Urbanized | 7/29/2016 9:45 | 7/31/2016 0:00 | 154 | 0.73 | 6.5 (5.8) | 0.6 - 45.6 | 0.27 | 314 (245) | 11 - 1020 |
| 972 | Highly Urbanized | 1/26/2016 14:15 | 1/27/2016 17:15 | 109 | 0.53 | 4.7 (5.4) | 0 - 26.4 | 0.47 | 457 (656) | 1 - 2985 |
| 972 | Highly Urbanized | 1/29/2016 1:45 | 1/30/2016 4:30 | 108 | 0.57 | 4.2 (3.8) | 0 - 20.2 | 0.43 | 447 (442) | 3 - 1524 |

Table 2. Movement characteristics of individual bursts of continuous coyote steps (15 minutes) in the Chicago Metropolitan Area from 2008 to 2017. We applied a three-state movement model to bursts to identify three movement behaviors, encamped, foraging, and traveling. Movements of coyotes persisting in natural urban landscapes could not be categorized into three ecologically-meaningful movement states.

| ID | Urban landscape | First location (m/d/y hh:mm) | Last location (m/d/y hh:mm) | Steps | Encamped | | | Foraging | | | Traveling | | |
| --- | --- | --- | --- | --- | --- | --- | --- | --- | --- | --- | --- | --- | --- |
|  |  |  |  |  | Time spent  (prop) | Average step-length (m; SD) | Step-length range (m) | Time spent  (prop) | Average step-length  (m; SD) | Step-length range (m) | Time spent  (prop) | Average step-length  (m; SD) | Step-length range (m) |
| 227 | Suburban | 8/21/2008 0:00 | 8/22/2008 23:45 | 192 | 0.55 | 7 (6.1) | 0.1 - 28.2 | 0.22 | 138 (139) | 3 - 727 | 0.23 | 539 (225) | 139 - 1031 |
| 434 | Suburban | 2/24/2010 1:00 | 2/28/2010 11:00 | 425 | 0.63 | 5.1 (4.6) | 0 - 26.7 | 0.36 | 139 (95) | 2 - 420 | 0.01 | 315 (26) | 290 - 341 |
| 434 | Suburban | 3/12/2010 1:00 | 3/15/2010 10:45 | 328 | 0.63 | 5.6 (5.6) | 0.2 - 37.9 | 0.34 | 122 (89) | 3 - 360 | 0.04 | 488 (162) | 249 - 699 |
| 434 | Suburban | 4/14/2010 1:00 | 4/18/2010 11:00 | 425 | 0.53 | 6 (5.5) | 0.1 - 33.4 | 0.36 | 143 (113) | 1 - 496 | 0.10 | 388 (156) | 190 - 1015 |
| 434 | Suburban | 5/12/2010 1:00 | 5/14/2010 10:45 | 232 | 0.59 | 6.5 (5.3) | 0 - 31.4 | 0.34 | 129 (93) | 2 - 398 | 0.08 | 494 (177) | 216 - 873 |
| 434 | Suburban | 6/18/2010 2:00 | 6/19/2010 15:15 | 150 | 0.57 | 6.7 (5.2) | 1 - 24.4 | 0.40 | 128 (102) | 2 - 402 | 0.03 | 519 (208) | 279 - 811 |
| 434 | Suburban | 6/20/2010 19:30 | 6/21/2010 20:15 | 100 | 0.68 | 5.9 (4.6) | 0.4 - 19.3 | 0.32 | 101 (91) | 1 - 399 | 0.00 | - | - |
| 854 | Suburban | 3/24/2016 0:00 | 3/30/2016 23:45 | 672 | 0.55 | 8.6 (7.1) | 0 - 35.9 | 0.19 | 137 (136) | 1 - 609 | 0.25 | 496 (207) | 115 - 1030 |
| 854 | Suburban | 5/24/2016 0:00 | 5/25/2016 8:00 | 129 | 0.30 | 10.5 (6.3) | 1 - 23.9 | 0.32 | 89 (111) | 2 - 572 | 0.38 | 492 (218) | 195 - 1083 |
| 854 | Suburban | 5/25/2016 8:30 | 5/30/2016 23:45 | 542 | 0.54 | 7.9 (6) | 0.1 - 31.1 | 0.23 | 120 (116) | 1 - 595 | 0.23 | 556 (222) | 175 - 1099 |
| 854 | Suburban | 7/24/2016 0:00 | 7/30/2016 23:45 | 672 | 0.54 | 8 (6.7) | 0.1 - 34.6 | 0.20 | 128 (135) | 0 - 631 | 0.26 | 536 (243) | 108 - 1287 |
| 854 | Suburban | 11/24/2016 0:00 | 11/30/2016 23:45 | 672 | 0.50 | 6.9 (5.8) | 0.2 - 39.4 | 0.25 | 135 (127) | 1 - 729 | 0.25 | 575 (226) | 178 - 1235 |
| 854 | Suburban | 1/24/2017 0:15 | 1/30/2017 23:45 | 671 | 0.60 | 5.3 (4.7) | 0.1 - 30.5 | 0.14 | 135 (92) | 1 - 427 | 0.26 | 537 (194) | 108 - 1100 |
| 750 | Highly Urbanized | 11/2/2014 20:15 | 11/4/2014 23:45 | 207 | 0.68 | 6.7 (5.4) | 0.4 - 22.9 | 0.18 | 128 (101) | 9 - 537 | 0.14 | 529 (253) | 144 - 1076 |
| 855 | Highly Urbanized | 12/9/2014 0:00 | 12/12/2014 23:45 | 384 | 0.46 | 5.5 (5.2) | 0.1 - 25.1 | 0.24 | 150 (116) | 4 - 721 | 0.3 | 682 (388) | 121 - 1784 |
| 855 | Highly Urbanized | 2/9/2015 1:30 | 2/12/2015 7:00 | 311 | 0.53 | 5.6 (4.6) | 0 - 29.3 | 0.21 | 129 (134) | 0 - 610 | 0.25 | 750 (404) | 124 - 1618 |
| 855 | Highly Urbanized | 5/9/2015 0:00 | 5/11/2015 1:30 | 199 | 0.59 | 5.2 (4.4) | 0.4 - 21 | 0.16 | 143 (153) | 0 - 724 | 0.25 | 621 (341) | 134 - 1534 |
| 855 | Highly Urbanized | 5/11/2015 2:00 | 5/13/2015 0:00 | 185 | 0.68 | 7.7 (5.5) | 0.1 - 28.3 | 0.16 | 100 (106) | 5 - 508 | 0.16 | 833 (572) | 289 - 2592 |
| 855 | Highly Urbanized | 7/9/2015 0:00 | 7/12/2015 23:45 | 384 | 0.5 | 6.5 (4.7) | 0.3 - 31.7 | 0.24 | 153 (134) | 2 - 609 | 0.26 | 981 (607) | 118 - 2556 |
| 855 | Highly Urbanized | 9/9/2015 0:00 | 9/12/2015 6:45 | 316 | 0.52 | 8.5 (7.1) | 0.5 - 33 | 0.22 | 94 (109) | 1 - 583 | 0.25 | 750 (396) | 47 - 1965 |
| 866 | Highly Urbanized | 3/9/2015 0:00 | 3/12/2015 23:45 | 384 | 0.67 | 7.3 (4.8) | 0.4 - 26.5 | 0.21 | 120 (117) | 4 - 524 | 0.13 | 484 (281) | 110 - 1747 |
| 866 | Highly Urbanized | 5/9/2015 1:00 | 5/12/2015 14:45 | 344 | 0.59 | 6 (5.8) | 0.1 - 35.3 | 0.19 | 101 (89) | 8 - 437 | 0.21 | 619 (304) | 53 - 1432 |
| 866 | Highly Urbanized | 7/9/2015 0:00 | 7/11/2015 15:45 | 256 | 0.55 | 8.2 (6.5) | 0.2 - 35.1 | 0.3 | 130 (109) | 7 - 458 | 0.15 | 486 (299) | 69 - 1399 |
| 866 | Highly Urbanized | 7/11/2015 16:15 | 7/12/2015 23:45 | 127 | 0.65 | 7.2 (5.2) | 0.8 - 24.7 | 0.2 | 115 (162) | 8 - 603 | 0.14 | 613 (306) | 50 - 1227 |
| 866 | Highly Urbanized | 9/9/2015 0:00 | 9/12/2015 9:00 | 325 | 0.6 | 7.4 (5.5) | 0 - 37.2 | 0.18 | 81 (88) | 2 - 533 | 0.22 | 589 (343) | 62 - 1455 |
| 885 | Highly Urbanized | 5/9/2015 8:00 | 5/11/2015 9:45 | 200 | 0.45 | 5.2 (4.1) | 0.3 - 22.2 | 0.14 | 132 (96) | 3 - 359 | 0.42 | 586 (309) | 87 - 1428 |
| 885 | Highly Urbanized | 7/9/2015 0:00 | 7/10/2015 14:30 | 155 | 0.6 | 6.3 (5.6) | 0.2 - 29.2 | 0.15 | 135 (104) | 29 - 426 | 0.25 | 642 (314) | 45 - 1396 |
| 885 | Highly Urbanized | 7/10/2015 15:00 | 7/12/2015 20:30 | 215 | 0.66 | 6.5 (4.3) | 0.9 - 23.2 | 0.11 | 89 (76) | 3 - 310 | 0.23 | 569 (315) | 73 - 1308 |
| 885 | Highly Urbanized | 9/10/2015 16:45 | 9/12/2015 1:15 | 131 | 0.68 | 6.2 (6.1) | 0.1 - 27.6 | 0.12 | 137 (156) | 12 - 611 | 0.2 | 574 (291) | 103 - 1238 |
| 970 | Highly Urbanized | 1/24/2016 0:00 | 1/30/2016 23:45 | 672 | 0.65 | 5.5 (5.2) | 0.1 - 37.4 | 0.15 | 133 (108) | 2 - 530 | 0.19 | 529 (313) | 67 - 1401 |
| 970 | Highly Urbanized | 5/24/2016 0:00 | 5/30/2016 19:00 | 653 | 0.57 | 7.9 (6.4) | 0.1 - 31 | 0.26 | 100 (97) | 0 - 511 | 0.17 | 490 (288) | 44 - 1537 |
| 970 | Highly Urbanized | 7/25/2016 23:30 | 7/29/2016 17:15 | 360 | 0.56 | 7.5 (6.1) | 0.4 - 29.7 | 0.31 | 107 (102) | 0 - 477 | 0.13 | 462 (237) | 88 - 939 |
| 970 | Highly Urbanized | 7/29/2016 18:45 | 7/30/2016 23:45 | 117 | 0.58 | 8.7 (6.2) | 0.5 - 32.9 | 0.27 | 97 (113) | 5 - 464 | 0.15 | 460 (214) | 122 - 814 |
| 970 | Highly Urbanized | 11/24/2016 0:00 | 11/30/2016 23:45 | 672 | 0.55 | 5.4 (4.7) | 0 - 30.5 | 0.25 | 148 (132) | 3 - 830 | 0.2 | 565 (275) | 106 - 1399 |
| 971 | Highly Urbanized | 1/24/2016 0:00 | 1/28/2016 9:45 | 424 | 0.54 | 5.4 (4.7) | 0 - 31.5 | 0.26 | 133 (114) | 0 - 510 | 0.2 | 666 (341) | 61 - 1512 |
| 971 | Highly Urbanized | 5/23/2016 23:45 | 5/25/2016 20:45 | 181 | 0.71 | 8 (6.2) | 0.5 - 34.4 | 0.18 | 126 (128) | 3 - 637 | 0.11 | 530 (329) | 81 - 1191 |
| 971 | Highly Urbanized | 5/27/2016 12:00 | 5/30/2016 23:45 | 336 | 0.72 | 8 (6.2) | 0.5 - 31 | 0.14 | 97 (90) | 2 - 326 | 0.13 | 516 (315) | 143 - 1596 |
| 971 | Highly Urbanized | 7/29/2016 9:45 | 7/31/2016 0:00 | 154 | 0.72 | 6.2 (4.5) | 0.6 - 26.4 | 0.13 | 110 (89) | 5 - 374 | 0.15 | 467 (218) | 151 - 1020 |
| 972 | Highly Urbanized | 1/26/2016 14:15 | 1/27/2016 17:15 | 109 | 0.53 | 4.7 (5.4) | 0 - 26.4 | 0.28 | 141 (105) | 1 - 375 | 0.18 | 932 (837) | 77 - 2985 |
| 972 | Highly Urbanized | 1/29/2016 1:45 | 1/30/2016 4:30 | 108 | 0.57 | 4.2 (3.8) | 0 - 20.2 | 0.21 | 112 (81) | 3 - 293 | 0.21 | 782 (398) | 158 - 1524 |

Appendix 3. Plots distinguishing the predicted step lengths and turning angles and the observed step lengths of different movement states


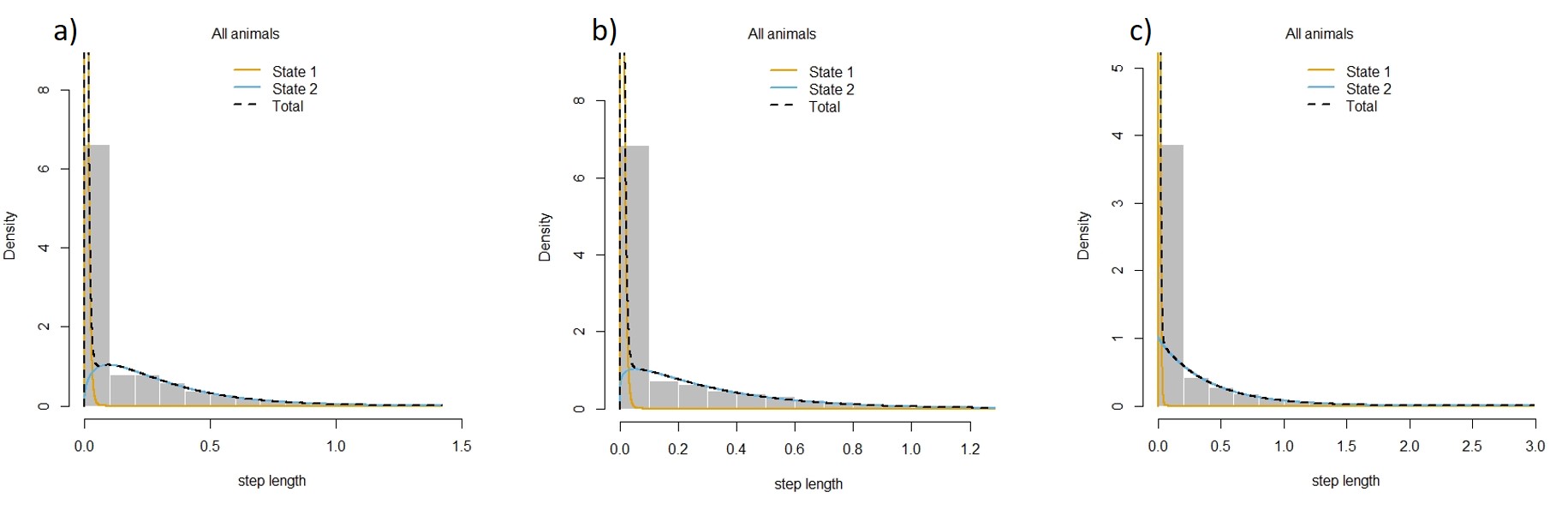


Figure 1. Predicted density of step-lengths (m) of the two-state movement model for coyotes in natural fragments (a), suburban landscapes (b), and highly urbanized landscapes (c). State 1 (orange) is typical of encamped behavior and state 2 (blue) is typical of moving behavior.


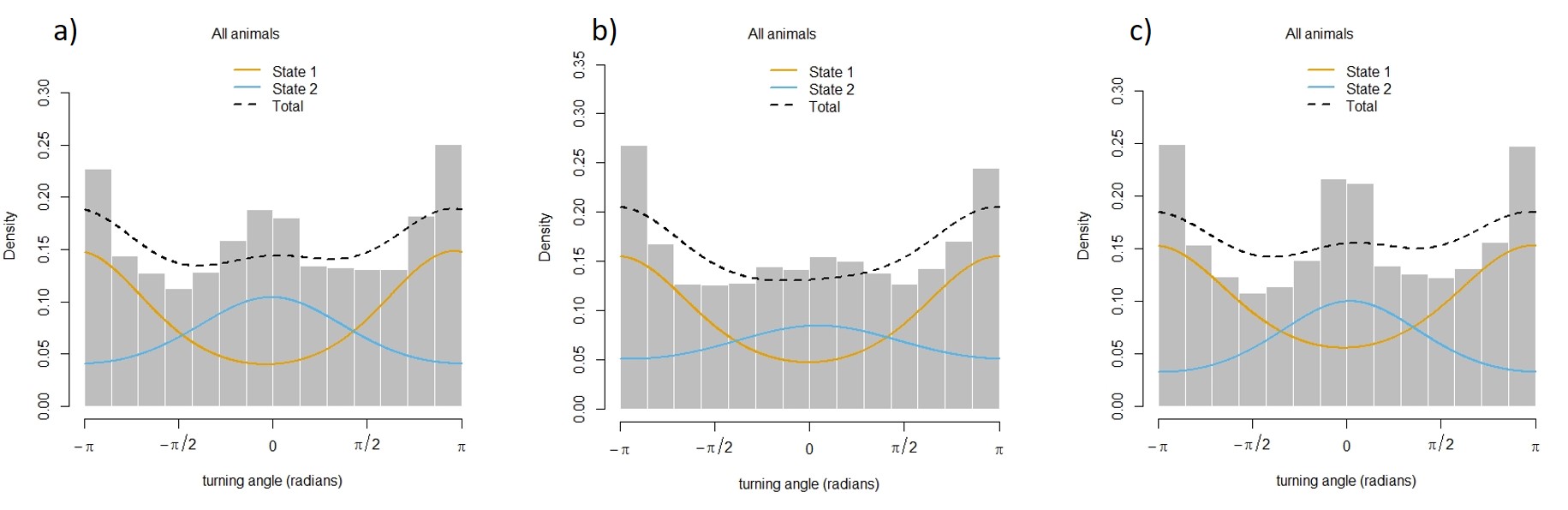
Figure 2. Predicted density of turning angles of the two-state movement model for coyotes in natural fragments (a), suburban landscapes (b), and highly urbanized landscapes (c). State 1 (orange) is typical of encamped behavior and state 2 (blue) is typical of moving behavior.


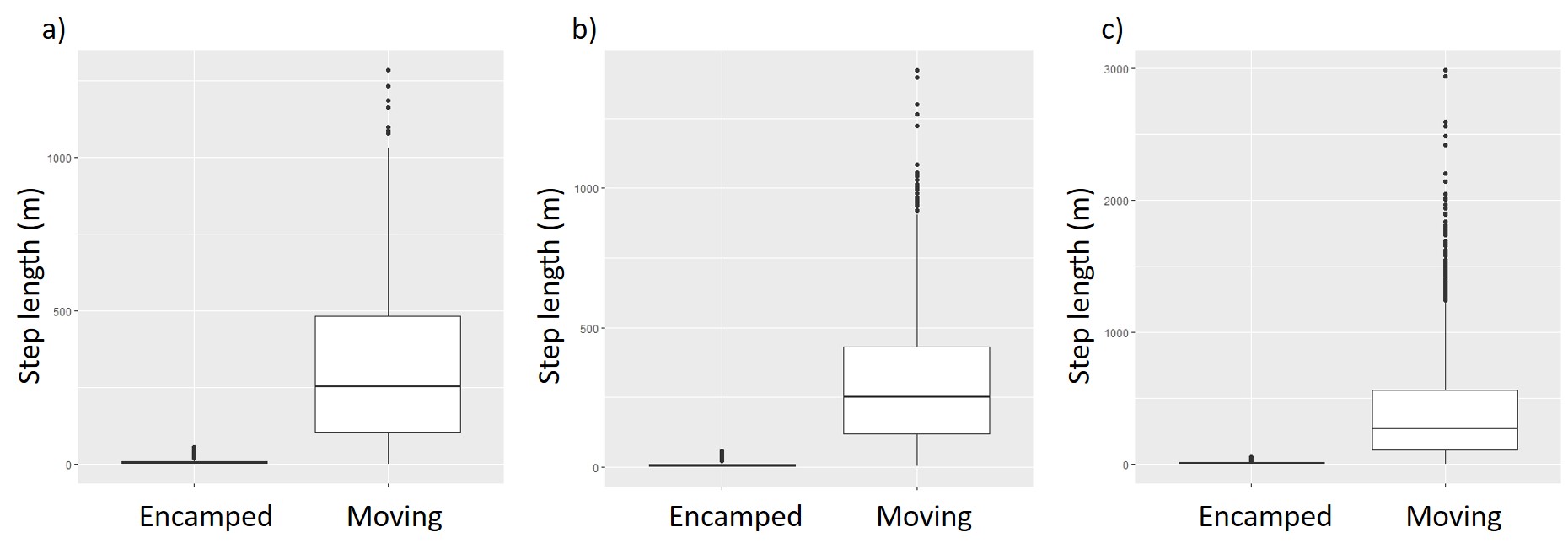
Figure 3. Distribution of observed step-lengths from the two-state movement model (encamped and moving) for coyotes in natural fragments (a), suburban landscapes (b), and highly urbanized landscapes (c).


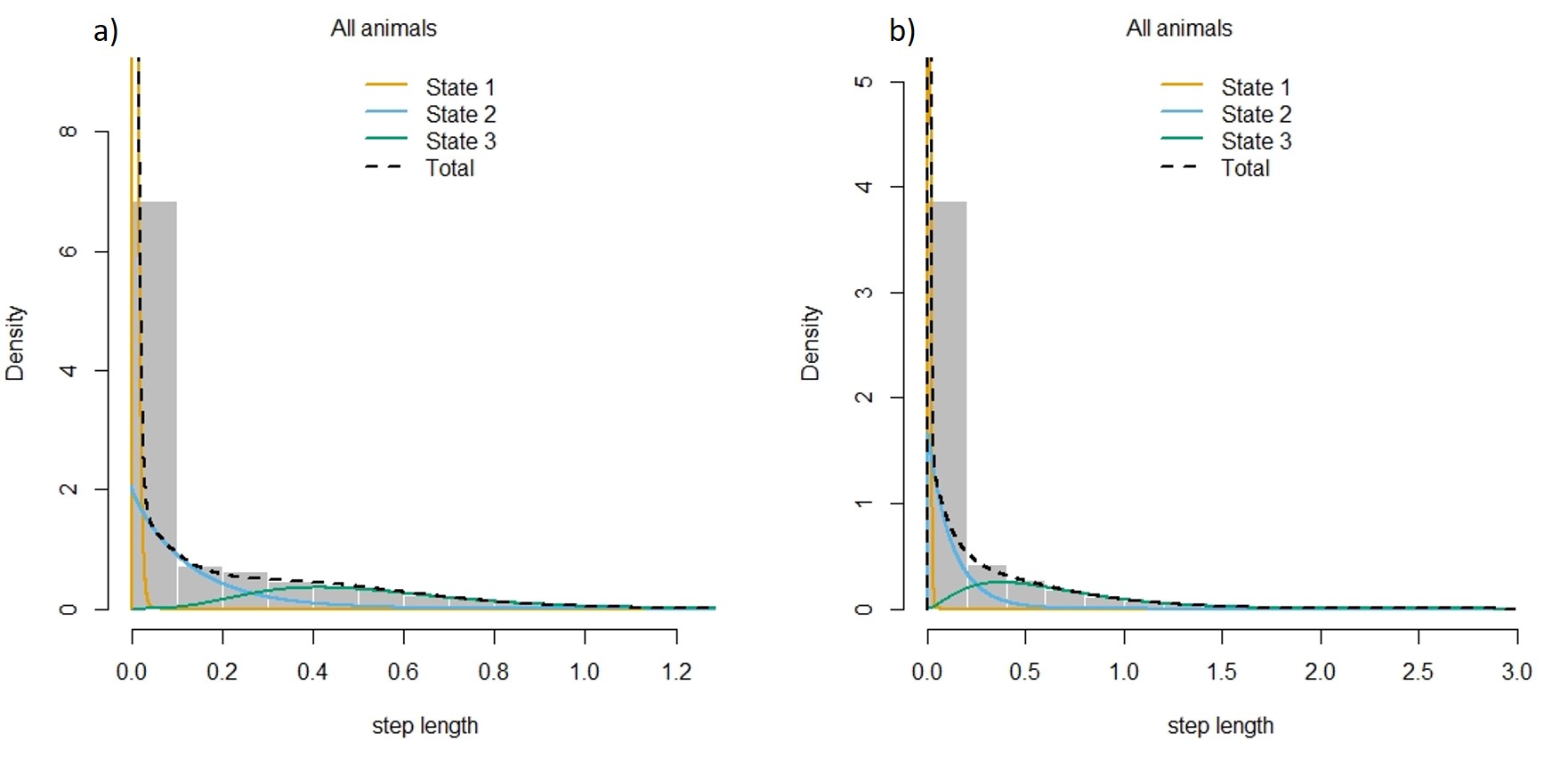
Figure 4. Predicted density of step-lengths (m) of the three-state movement model for coyotes in suburban landscapes (a), and highly urbanized landscapes (b). State 1 (orange) is typical of encamped behavior, state 2 (blue) is typical of foraging behavior, and state 3 (green) is typical of traveling behavior.


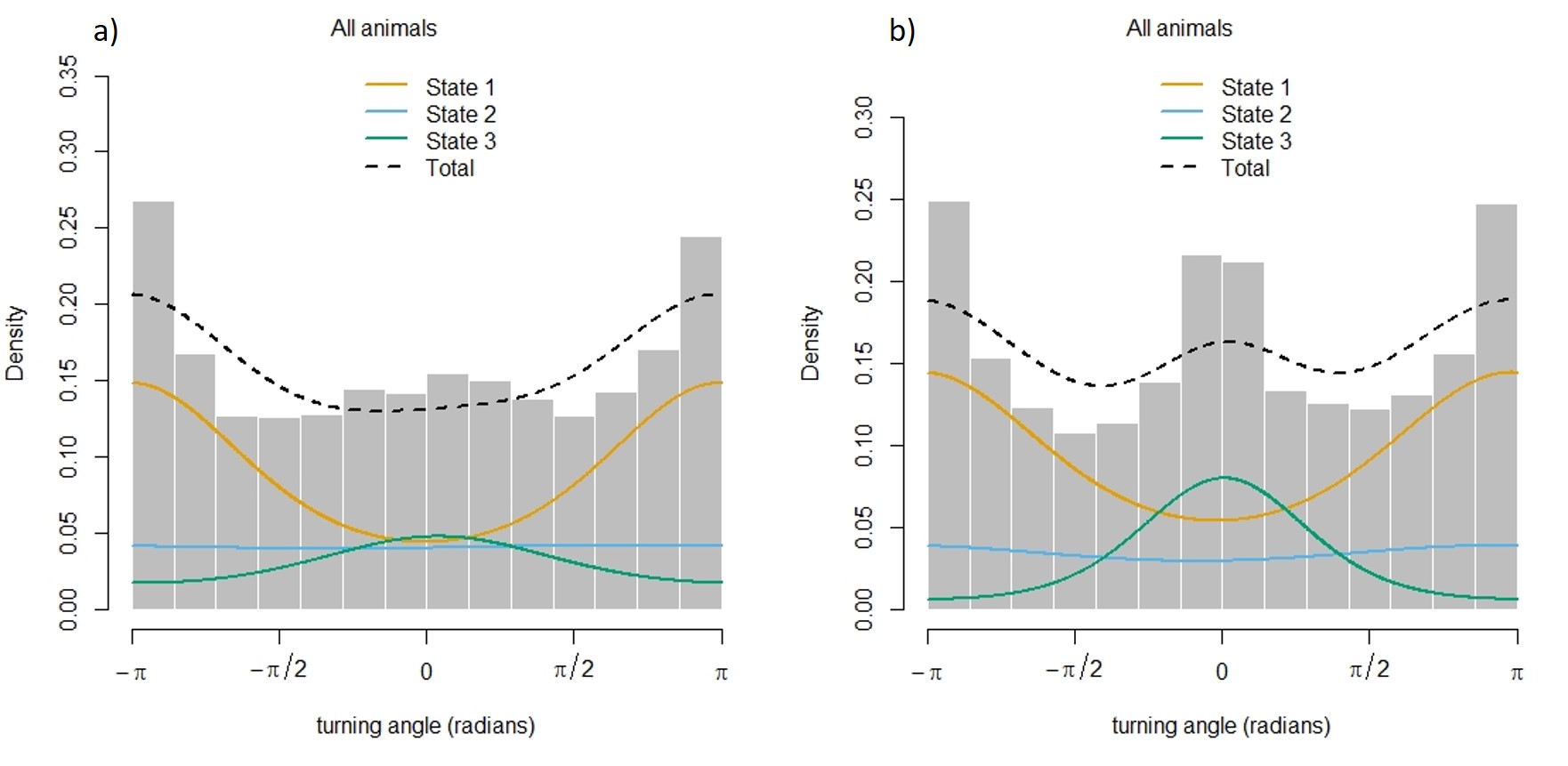
Figure 5. Predicted density of turning angles of the three-state movement model for coyotes in suburban landscapes (a), and highly urbanized landscapes (b). State 1 (orange) is typical of encamped behavior, state 2 (blue) is typical of foraging behavior, and state 3 (green) is typical of traveling behavior.


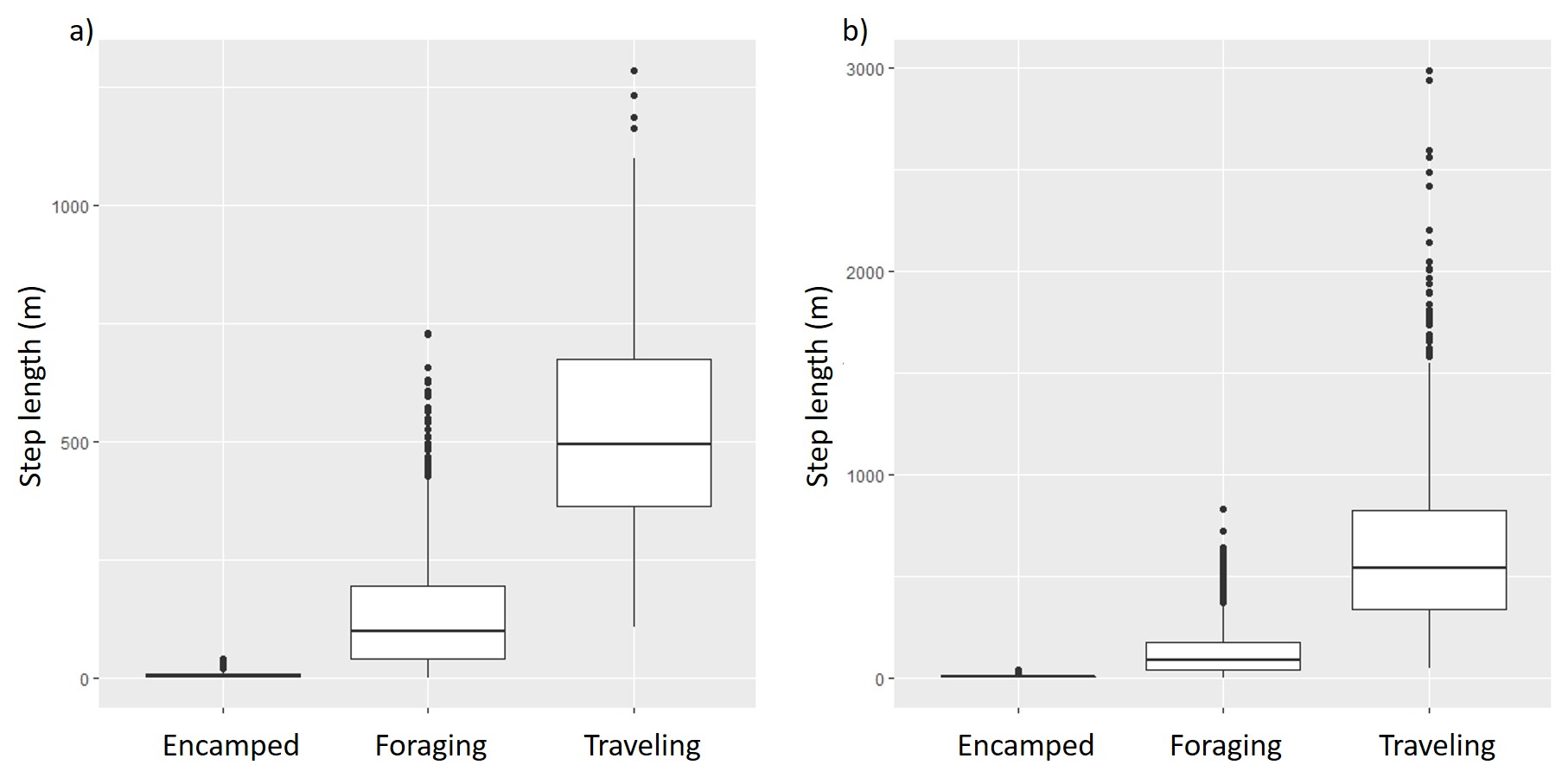
Figure 6. Distribution of observed step-lengths from the three-state movement model (encamped, foraging, traveling) for coyotes in suburban landscapes (a), and highly urbanized landscapes (b).

Appendix 4. Individual differences in space use and movement behavior of coyotes in the Chicago Metropolitan Area

Table 3. Home range size, complexity, and predicted movement behavior of individual coyotes (*Canis latrans*) within the Chicago metropolitan area, USA from 2008 – 2017.

| ID | Average percent of developed imperviousness | Number of days^a^ | Home range size^b^ (km^2^) | Home range complexity^c^ | Number of 15 min. segments | Encamped | | Foraging^d^ | | Moving/Traveling^d^ | |
| --- | --- | --- | --- | --- | --- | --- | --- | --- | --- | --- | --- |
|  |  |  |  |  |  | Average step-length  (m; SD) | Time spent  (prop) | Average  step-length  (m; SD) | Time spent  (prop) | Average  step-length  (m; SD) | Time spent  (prop) |
| 362 | 2 | 297 | 2.22 | 0.35 | - | - | - | - | - | - | - |
| 672 | 3 | 238 | 2.61 | 0.14 | 585 | 13.5 (10) | 0.44 | - | - | 255 (190) | 0.56 |
| 164 | 4.4 | 157 | 2.90 | 0.09 | - | - | - | - | - | - | - |
| 448 | 2.7 | 252 | 3.15 | 0.17 | - | - | - | - | - | - | - |
| 370 | 1.3 | 365 | 3.23 | 0.20 | 385 | 8.1 (6.3) | 0.65 | - | - | 287 (182) | 0.35 |
| 678 | 3.8 | 181 | 3.86 | 0.27 | 643 | 5.5 (6.7) | 0.57 | - | - | 311 (264) | 0.43 |
| 695 | 16.3 | 212 | 4.73 | 0.16 | 698 | 5 (7.5) | 0.62 | - | - | 373 (271) | 0.38 |
| 434 | 25.9 | 129 | 0.27 | 0.46 | 1660 | 5.8 (5.2) | 0.60 | 132 (99) | 0.36 | 431 (169) | 0.05 |
| 740 | 23.5 | 259 | 0.46 | 0.32 | - | - | - | - | - | - | - |
| 321 | 29.9 | 424 | 0.72 | 0.29 | - | - | - | - | - | - | - |
| 854 | 27.5 | 383 | 1.38 | 0.51 | 3358 | 7.4 (6.2) | 0.54 | 128 (123) | 0.21 | 536 (220) | 0.26 |
| 308 | 23.2 | 114 | 1.75 | 0.62 | - | - | - | - | - | - | - |
| 227 | 31.5 | 314 | 2.90 | 0.56 | 192 | 7 (6.1) | 0.55 | 138 (139) | 0.22 | 539 (225) | 0.23 |
| 298 | 40.4 | 271 | 8.67 | 0.23 | - | - | - | - | - | - | - |
| 970 | 78.2 | 364 | 1.26 | 0.83 | 2474 | 6.5 (5.7) | 0.59 | 121 (113) | 0.24 | 520 (286) | 0.18 |
| 971 | 79.5 | 311 | 1.61 | 0.89 | 1095 | 6.9 (5.6) | 0.65 | 122 (110) | 0.19 | 583 (327) | 0.16 |
| 885 | 65.2 | 304 | 2.08 | 0.18 | 701 | 6.1 (5) | 0.59 | 122 (107) | 0.13 | 591 (308) | 0.28 |
| 748 | 60.6 | 110 | 3.41 | 0.76 | - | - | - | - | - | - | - |
| 744 | 72.1 | 376 | 3.76 | 0.56 | - | - | - | - | - | - | - |
| 750 | 78.3 | 251 | 5.24 | 0.69 | 207 | 6.7 (5.4) | 0.68 | 128 (101) | 0.18 | 529 (253) | 0.14 |
| 866 | 53.5 | 251 | 9.56 | 0.65 | 1436 | 7.2 (5.6) | 0.61 | 110 (110) | 0.21 | 563 (314) | 0.17 |
| 855 | 59.1 | 299 | 13.42 | 0.74 | 1779 | 6.5 (5.5) | 0.53 | 132 (126) | 0.22 | 775 (471) | 0.25 |
| 441 | 62.2 | 139 | 23.12 | 0.58 | - | - | - | - | - | - | - |

^a^ Number of days individual coyotes were monitored with GPS collars with fixes obtained every 7.5hrs.

^b^ We estimated home range size as the local convex hull (LoCoH) isopleth nearest to 95% with the adehabitatHR package (Calenge 2006) in R (R Core Team 2017).

^c^ We estimated complexity index using the formula (95% MCP – 95% LoCoH)/95% MCP, where MCP is the 95% minimum convex polygon

^d^ Foraging and traveling were indistinguishable for coyotes in natural fragments, thus we report values for the two-state movement model (encamped and moving). The characteristics of coyote moving behaviour in natural fragments is therefore not comparable to the traveling behavior of coyotes in the other urban landscapes
