# Supplementary material for: Behavioral responses by an apex predator to urbanization
Source: Behav Ecol. 2019 Mar 2;30(3):821–9. doi: 10.1093/beheco/arz019 (PMC6562302; doi:10.1093/beheco/arz019)
Supplement: arz019_suppl_Map_Appendix [file arz019_suppl_map_appendix.pdf]

Figure 1. The home range (95% local convex hull, solid black line), core range (50% local convex hull, small dashed black line), and 95% minimum convex polygon (large dashed purple line) of coyote 370 from April 2009 to April 2010 in Chicago, IL, USA this coyote lived in a natural fragment (average percent of developed imperviousness = 1.3%).

Figure 2. The home range (95% local convex hull, solid black line), core range (50% local convex hull, small dashed black line), and 95% minimum convex polygon (large dashed purple line) of coyote 362 from May 2014 to March 2015 in Chicago, IL, USA this coyote lived in a natural fragment (average percent of developed imperviousness = 2.0%).

Figure 3. The home range (95% local convex hull, solid black line), core range (50% local convex hull, small dashed black line), and 95% minimum convex polygon (large dashed purple line) of coyote 448 from December 2010 to August 2011 in Chicago, IL, USA this coyote lived in a natural fragment (average percent of developed imperviousness = 2.7%).

Figure 4. The home range (95% local convex hull, solid black line), core range (50% local convex hull, small dashed black line), and 95% minimum convex polygon (large dashed purple line) of coyote 672 from January 2013 to September 2013 in Chicago, IL, USA this coyote lived in a natural fragment (average percent of developed imperviousness = 3.0%).

Figure 5. The home range (95% local convex hull, solid black line), core range (50% local convex hull, small dashed black line), and 95% minimum convex polygon (large dashed purple line) of coyote 678 from April 2015 to October 2015 in Chicago, IL, USA this coyote lived in a natural fragment (average percent of developed imperviousness = 3.8%).

Figure 6. The home range (95% local convex hull, solid black line), core range (50% local convex hull, small dashed black line), and 95% minimum convex polygon (large dashed purple line) of coyote 164 from April 2013 to September 2013 in Chicago, IL, USA this coyote lived in a natural fragment (average percent of developed imperviousness = 4.4%).

Figure 7. The home range (95% local convex hull, solid black line), core range (50% local convex hull, small dashed black line), and 95% minimum convex polygon (large dashed purple line) of coyote 695 from October 2015 to May 2016 in Chicago, IL, USA this coyote lived in a natural fragment (average percent of developed imperviousness = 16.3%).

Figure 8. The home range (95% local convex hull, solid black line), core range (50% local convex hull, small dashed black line), and 95% minimum convex polygon (large dashed purple line) of coyote 308

from May 2009 to September 2009 in Chicago, IL, USA this coyote lived in a natural fragment (average percent of developed imperviousness = 23.2%).

Figure 9. The home range (95% local convex hull, solid black line), core range (50% local convex hull, small dashed black line), and 95% minimum convex polygon (large dashed purple line) of coyote 740 from June 2013 to March 2014 in Chicago, IL, USA this coyote lived in a natural fragment (average percent of developed imperviousness = 23.5%).

Figure 10. The home range (95% local convex hull, solid black line), core range (50% local convex hull, small dashed black line), and 95% minimum convex polygon (large dashed purple line) of coyote 434 from February 2010 to June 2010 in Chicago, IL, USA this coyote lived in a natural fragment (average percent of developed imperviousness = 25.9%).

Figure 11. The home range (95% local convex hull, solid black line), core range (50% local convex hull, small dashed black line), and 95% minimum convex polygon (large dashed purple line) of coyote 854 from January 2016 to February 2017 in Chicago, IL, USA this coyote lived in a natural fragment (average percent of developed imperviousness = 27.5%).

Figure 12. The home range (95% local convex hull, solid black line), core range (50% local convex hull, small dashed black line), and 95% minimum convex polygon (large dashed purple line) of coyote 321 from April 2008 to June 2009 in Chicago, IL, USA this coyote lived in a natural fragment (average percent of developed imperviousness = 29.9%).

Figure 13. The home range (95% local convex hull, solid black line), core range (50% local convex hull, small dashed black line), and 95% minimum convex polygon (large dashed purple line) of coyote 227 from May 2008 to April 2009 in Chicago, IL, USA this coyote lived in a natural fragment (average percent of developed imperviousness = 31.5%).

Figure 14. The home range (95% local convex hull, solid black line), core range (50% local convex hull, small dashed black line), and 95% minimum convex polygon (large dashed purple line) of coyote 298 from June 2008 to March 2009 in Chicago, IL, USA this coyote lived in a natural fragment (average percent of developed imperviousness = 40.4%).

Figure 15. The home range (95% local convex hull, solid black line), core range (50% local convex hull, small dashed black line), and 95% minimum convex polygon (large dashed purple line) of coyote 866 from January 2015 to September 2015 in Chicago, IL, USA this coyote lived in a natural fragment (average percent of developed imperviousness = 53.5%).

Figure 16. The home range (95% local convex hull, solid black line), core range (50% local convex hull, small dashed black line), and 95% minimum convex polygon (large dashed purple line) of coyote 855 from November 2014 to September 2015 in Chicago, IL, USA this coyote lived in a natural fragment (average percent of developed imperviousness = 59.1%).

Figure 17. The home range (95% local convex hull, solid black line), core range (50% local convex hull, small dashed black line), and 95% minimum convex polygon (large dashed purple line) of coyote 748 from February 2014 to June 2014 in Chicago, IL, USA this coyote lived in a natural fragment (average percent of developed imperviousness = 60.6%).

Figure 18. The home range (95% local convex hull, solid black line), core range (50% local convex hull, small dashed black line), and 95% minimum convex polygon (large dashed purple line) of coyote 441 from March 2010 to July 2010 in Chicago, IL, USA this coyote lived in a natural fragment (average percent of developed imperviousness = 62.2%).

Figure 19. The home range (95% local convex hull, solid black line), core range (50% local convex hull, small dashed black line), and 95% minimum convex polygon (large dashed purple line) of coyote 885 from May 2015 to March 2016 in Chicago, IL, USA this coyote lived in a natural fragment (average percent of developed imperviousness = 65.2%).

Figure 20. The home range (95% local convex hull, solid black line), core range (50% local convex hull, small dashed black line), and 95% minimum convex polygon (large dashed purple line) of coyote 744 from November 2013 to November 2014 in Chicago, IL, USA this coyote lived in a natural fragment (average percent of developed imperviousness = 72.1%).

Figure 21. The home range (95% local convex hull, solid black line), core range (50% local convex hull, small dashed black line), and 95% minimum convex polygon (large dashed purple line) of coyote 970 from December 2015 to December 2016 in Chicago, IL, USA this coyote lived in a natural fragment (average percent of developed imperviousness = 78.2%).

Figure 22. The home range (95% local convex hull, solid black line), core range (50% local convex hull, small dashed black line), and 95% minimum convex polygon (large dashed purple line) of coyote 750 from March 2014 to November 2014 in Chicago, IL, USA this coyote lived in a natural fragment (average percent of developed imperviousness = 78.3%).

Figure 23. The home range (95% local convex hull, solid black line), core range (50% local convex hull, small dashed black line), and 95% minimum convex polygon (large dashed purple line) of coyote 971 from January 2016 to November 2016 in Chicago, IL, USA this coyote lived in a natural fragment (average percent of developed imperviousness = 79.5%).

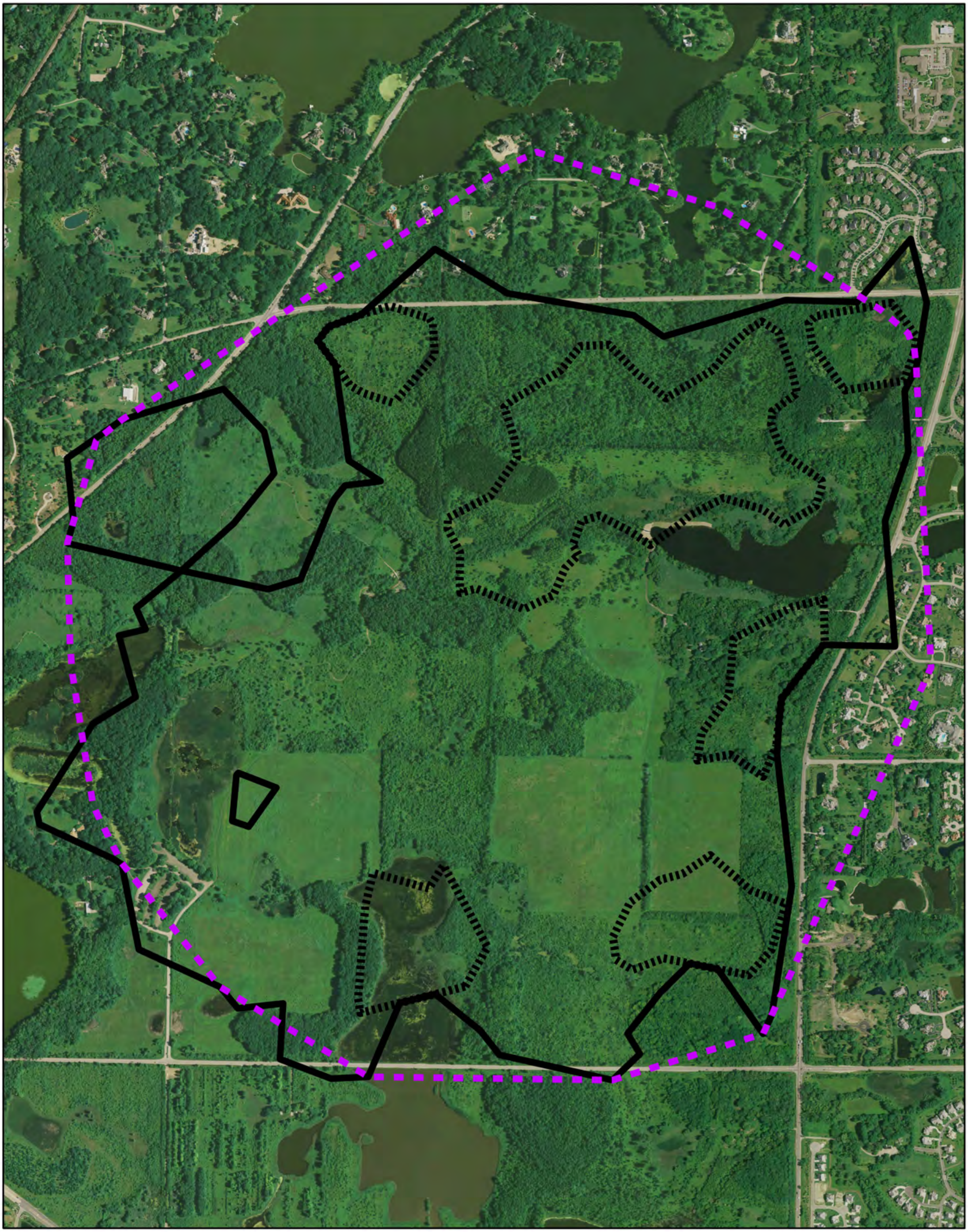

Figure 1

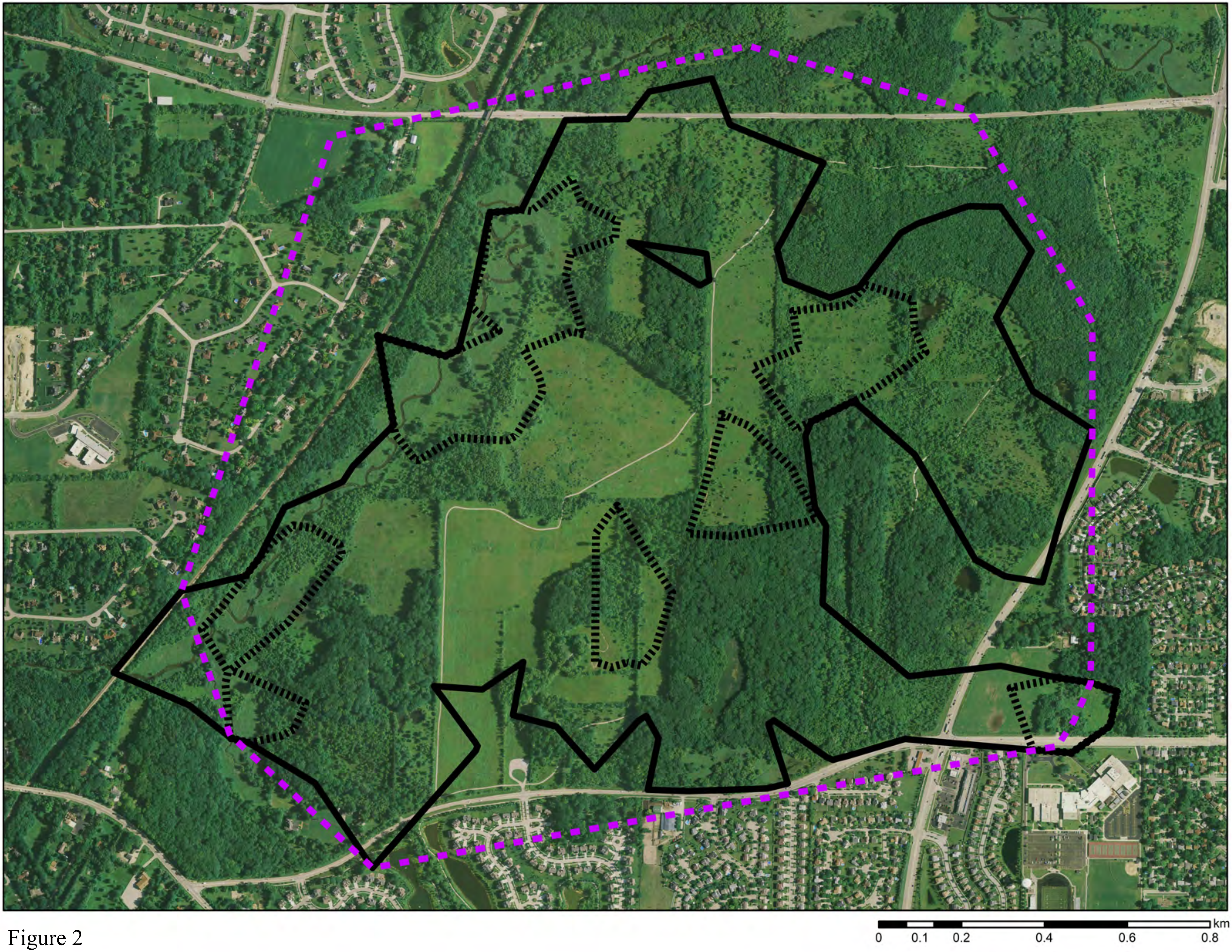

Figure 2

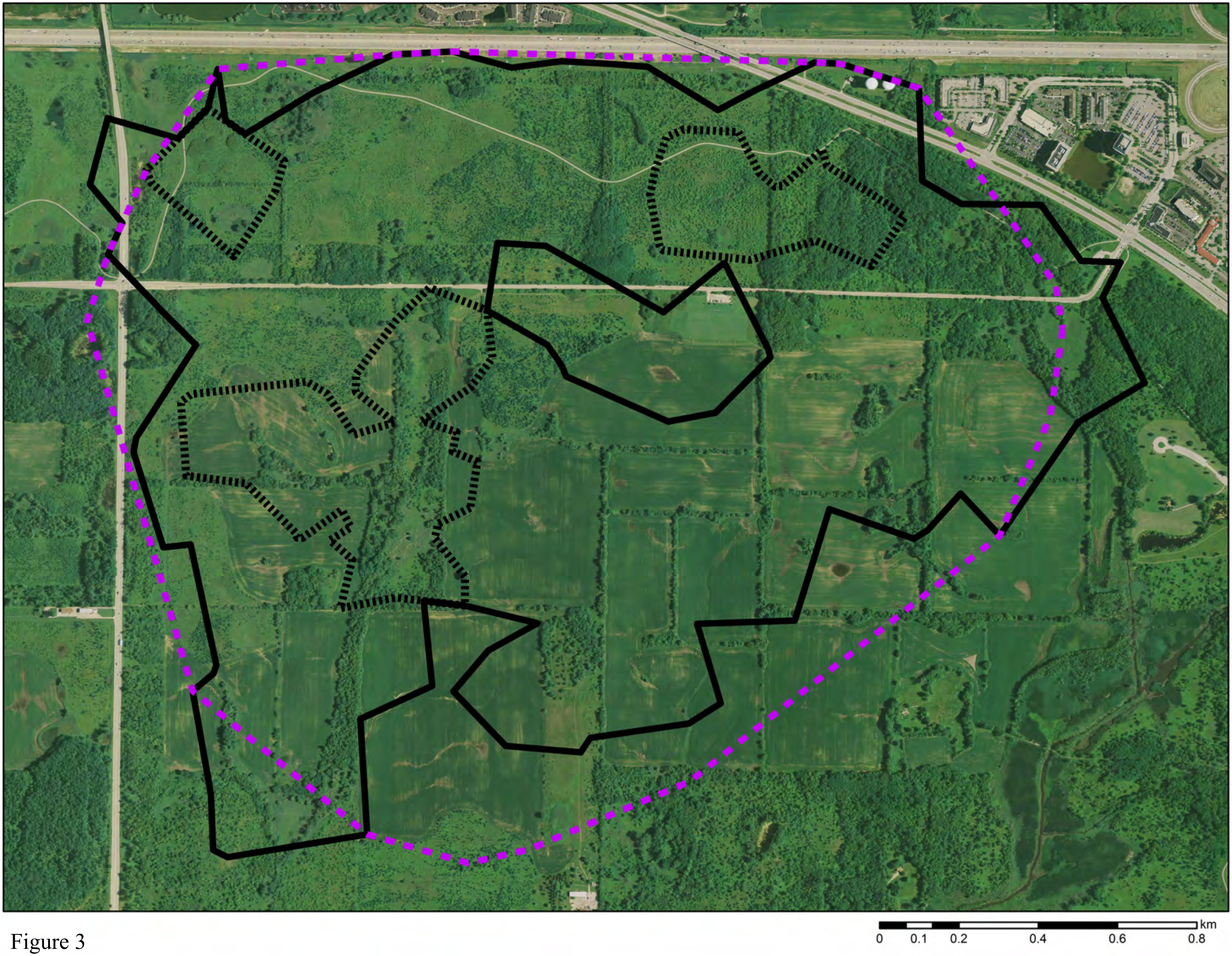

Figure 3

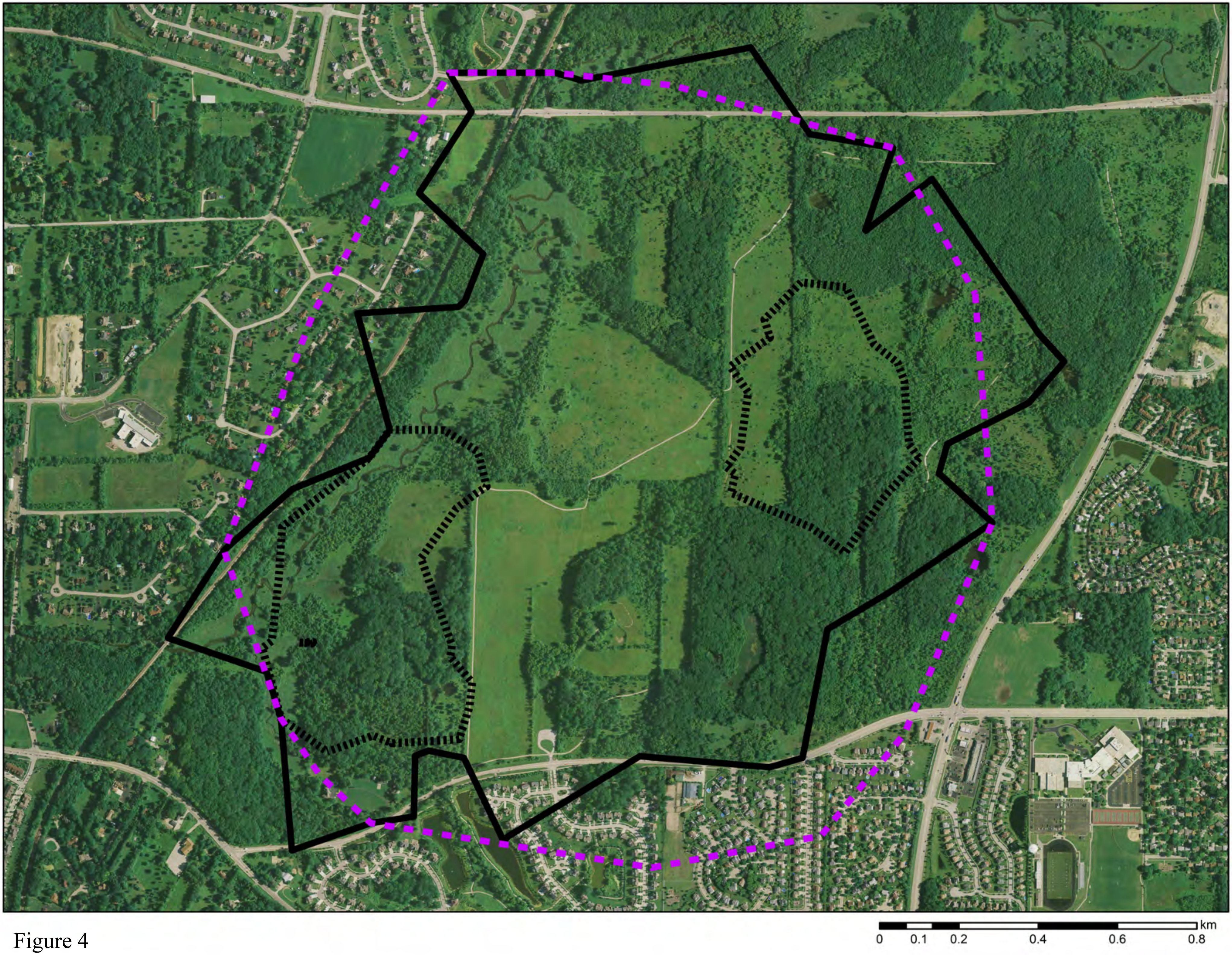

Figure 4

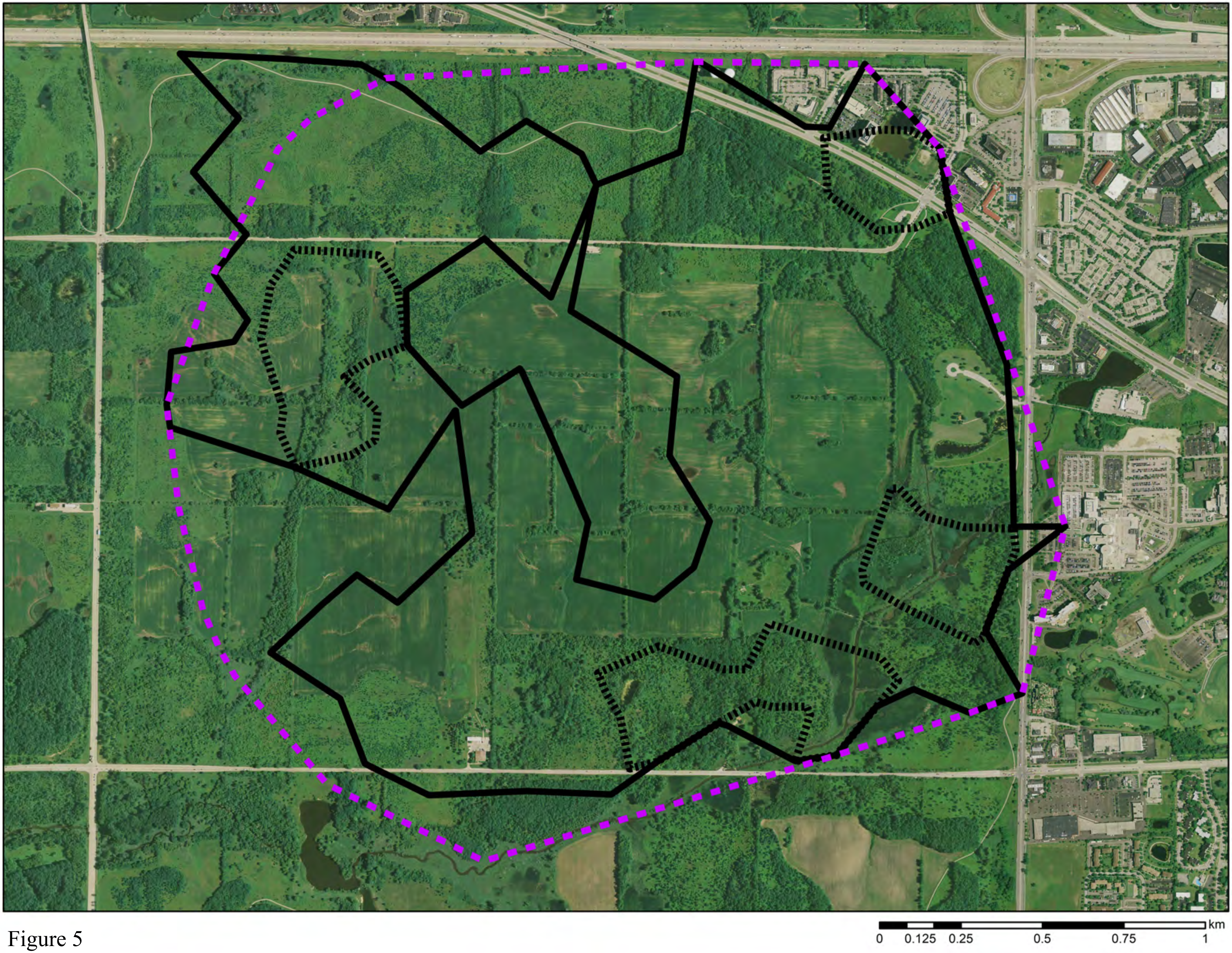

Figure 5

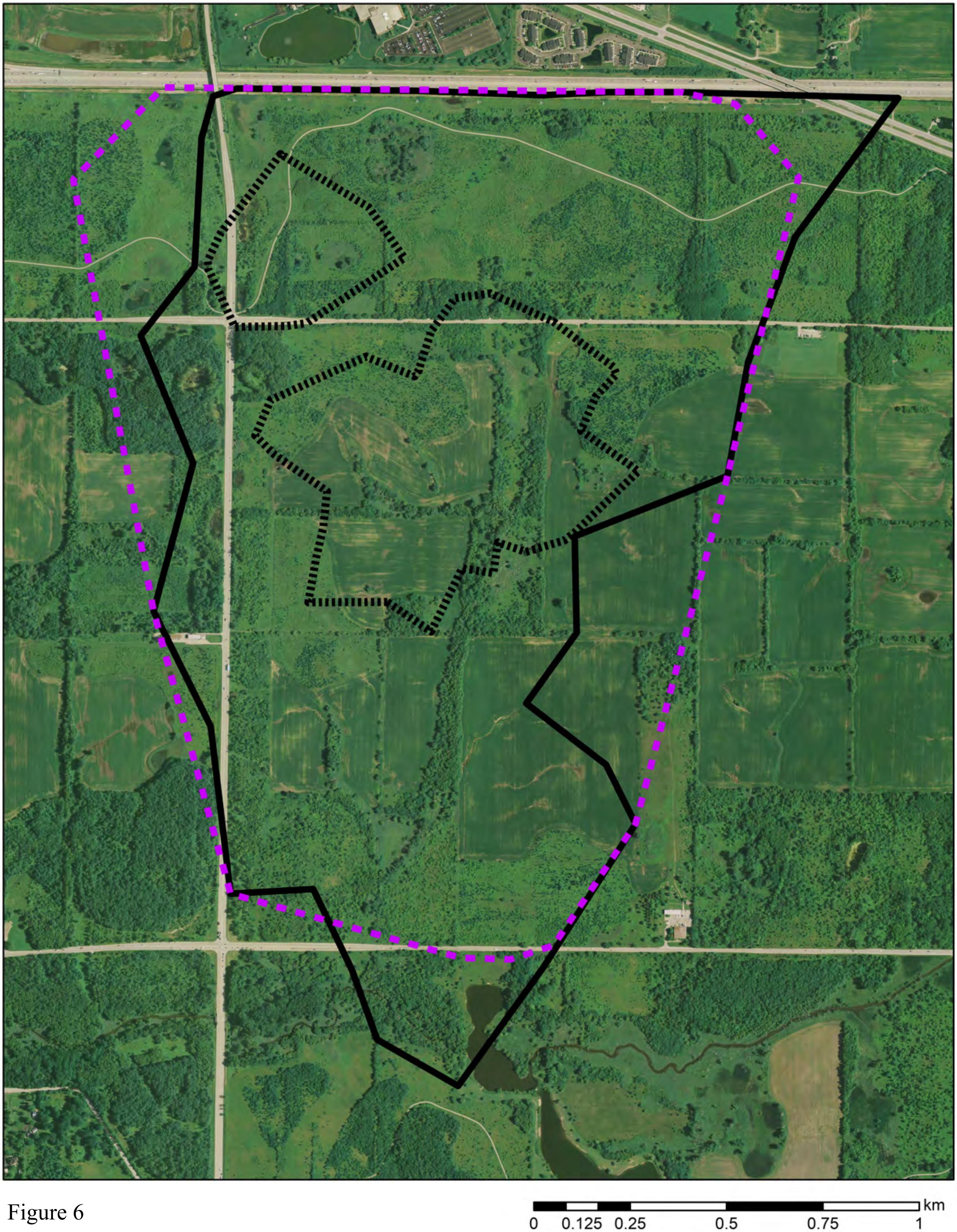

Figure 6

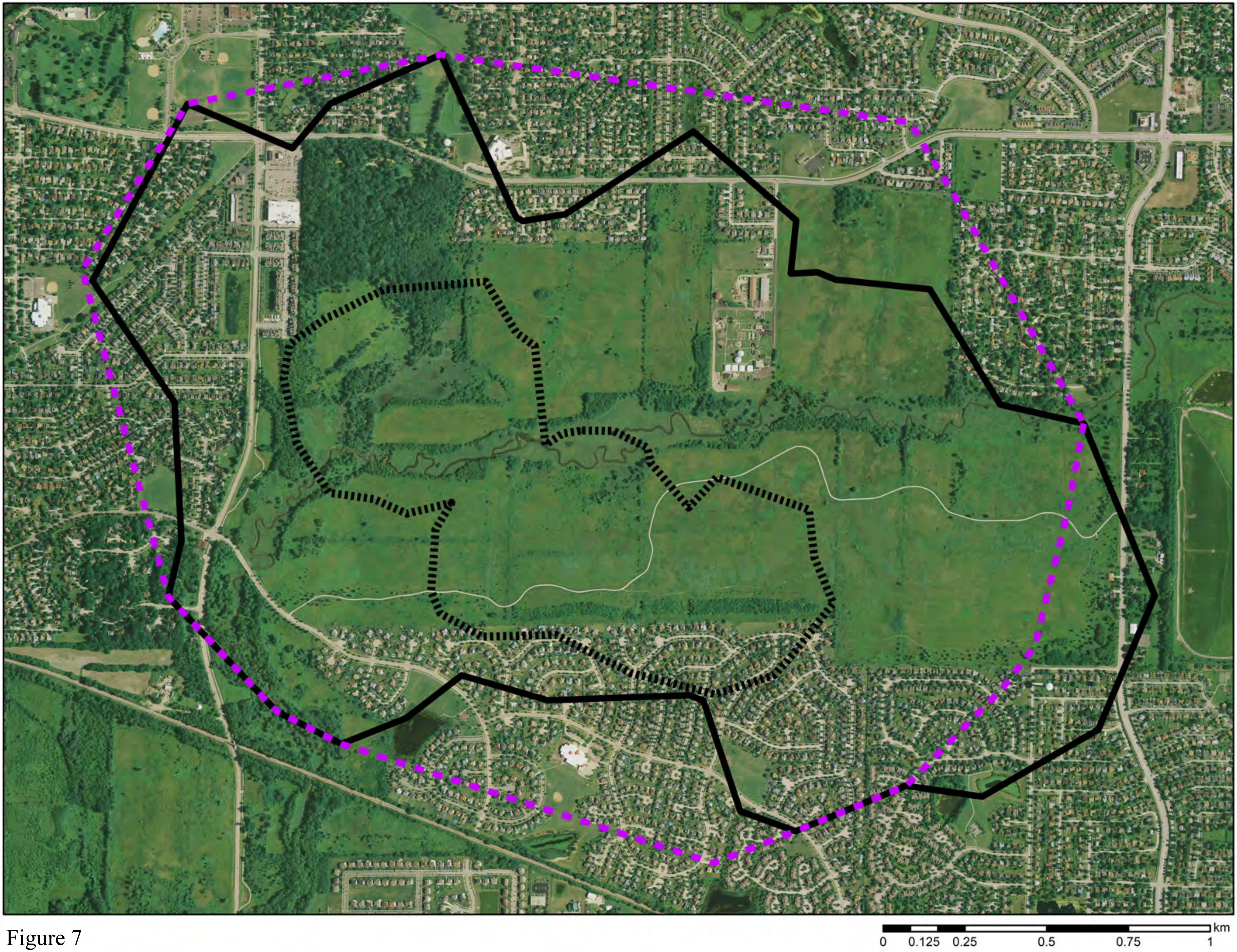

Figure 7

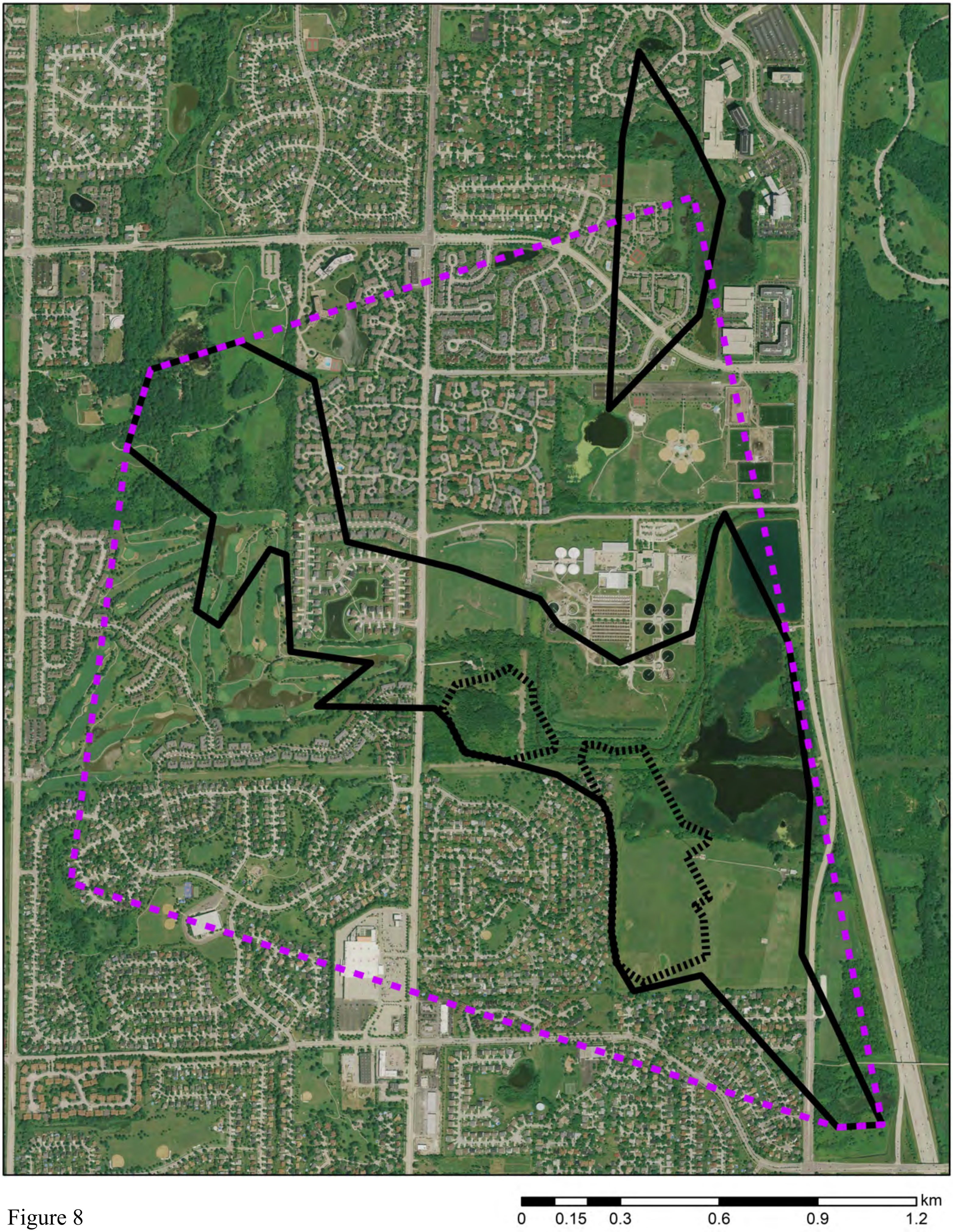

Figure 8

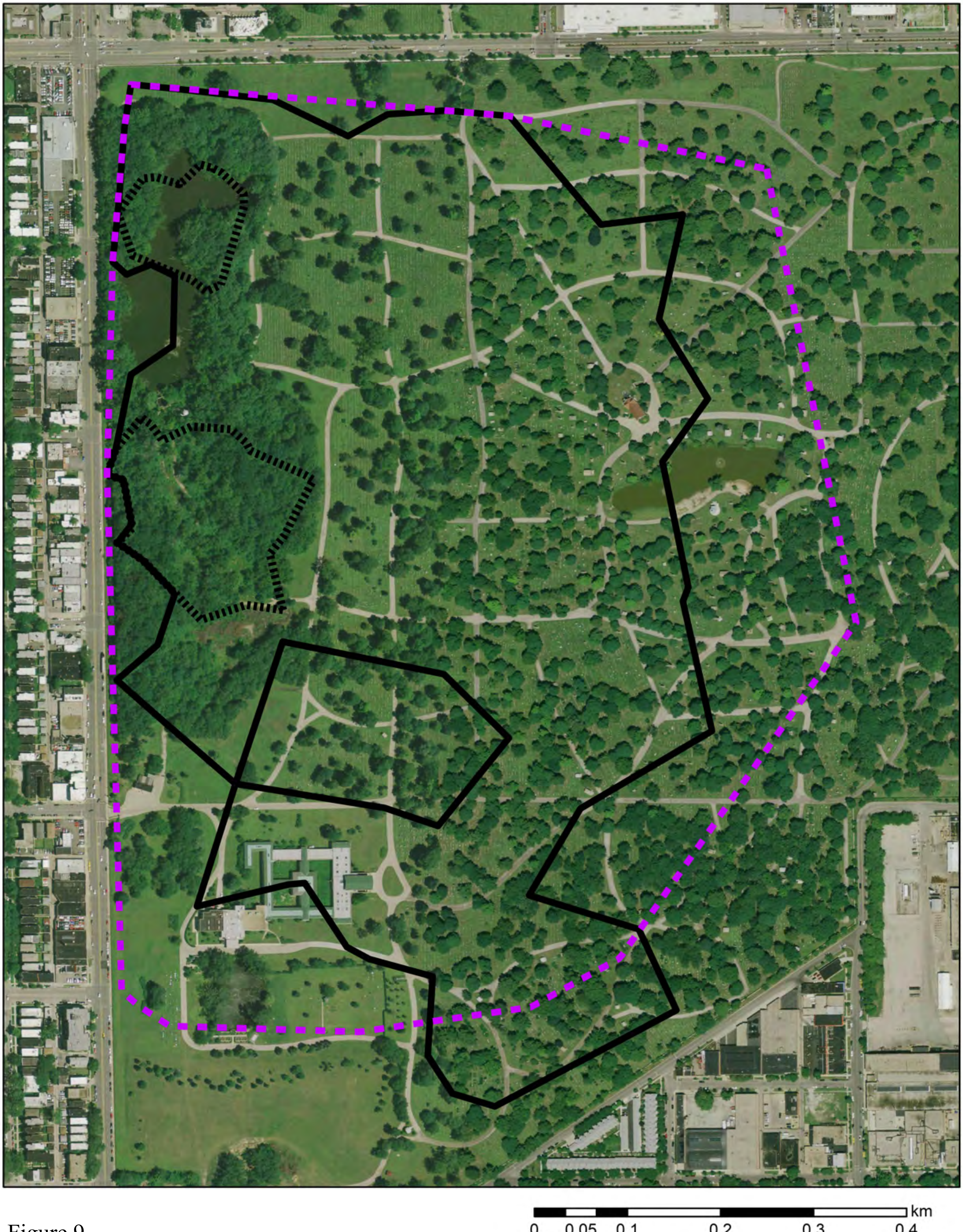

Figure 9

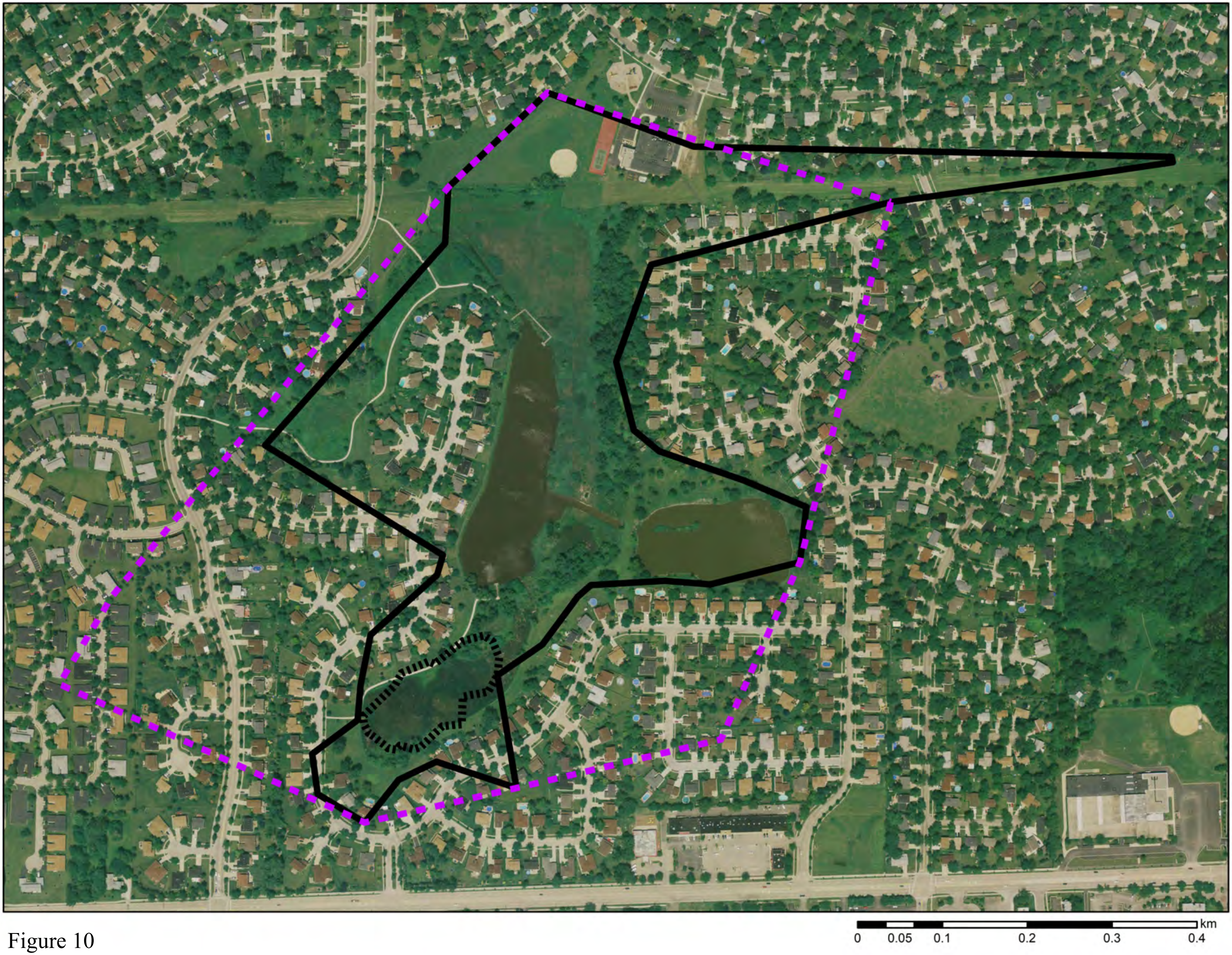

Figure 10

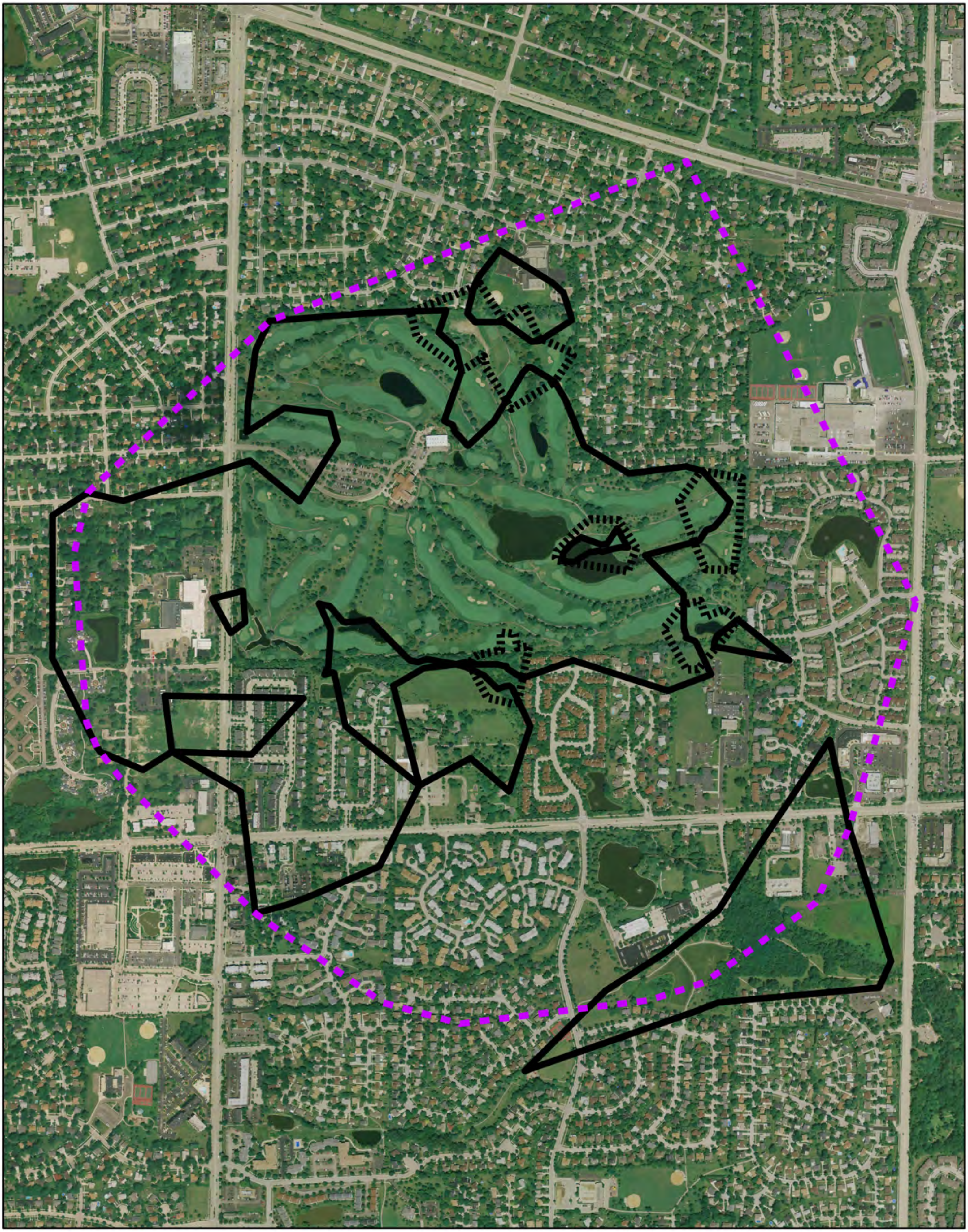

Figure 11

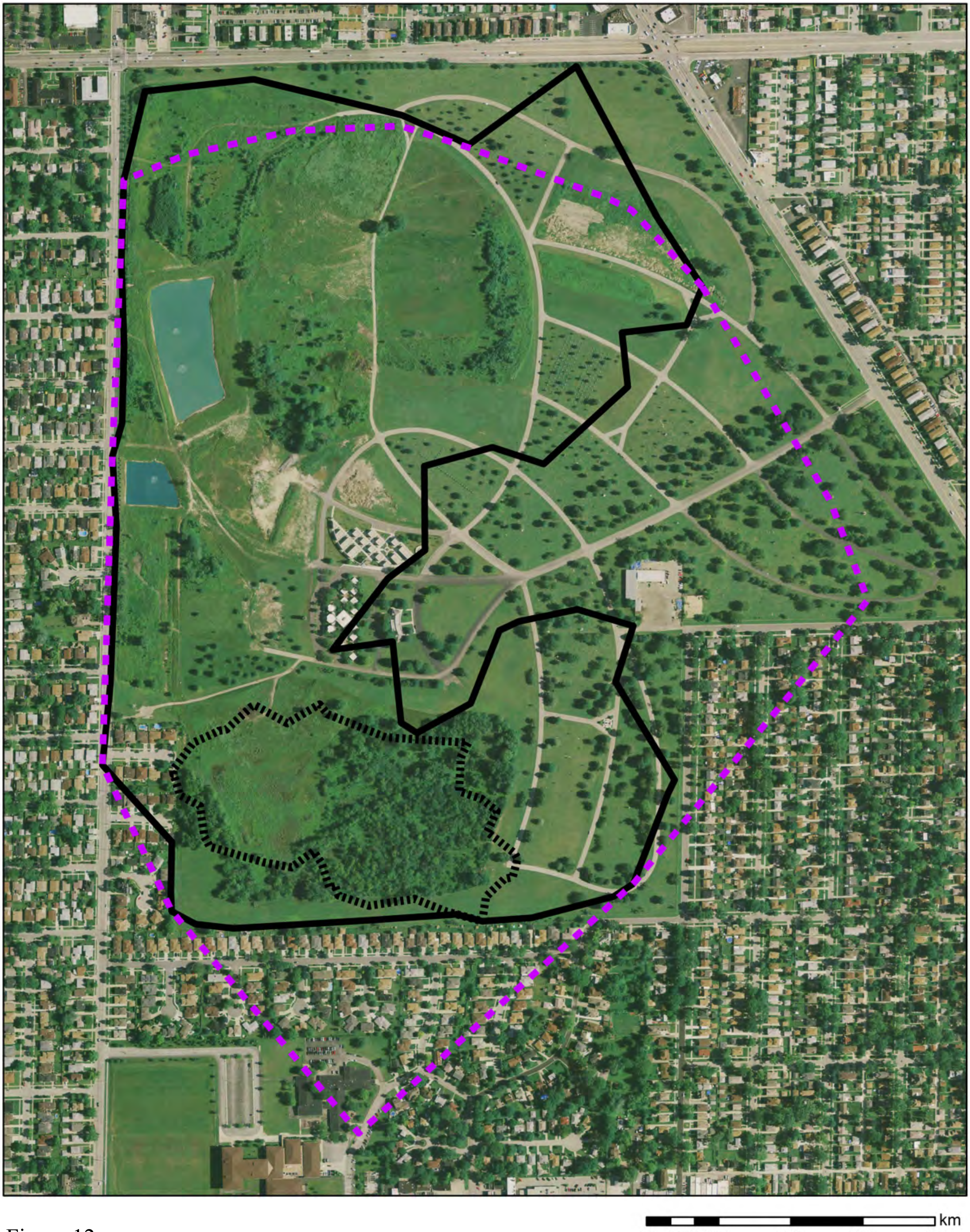

Figure 12

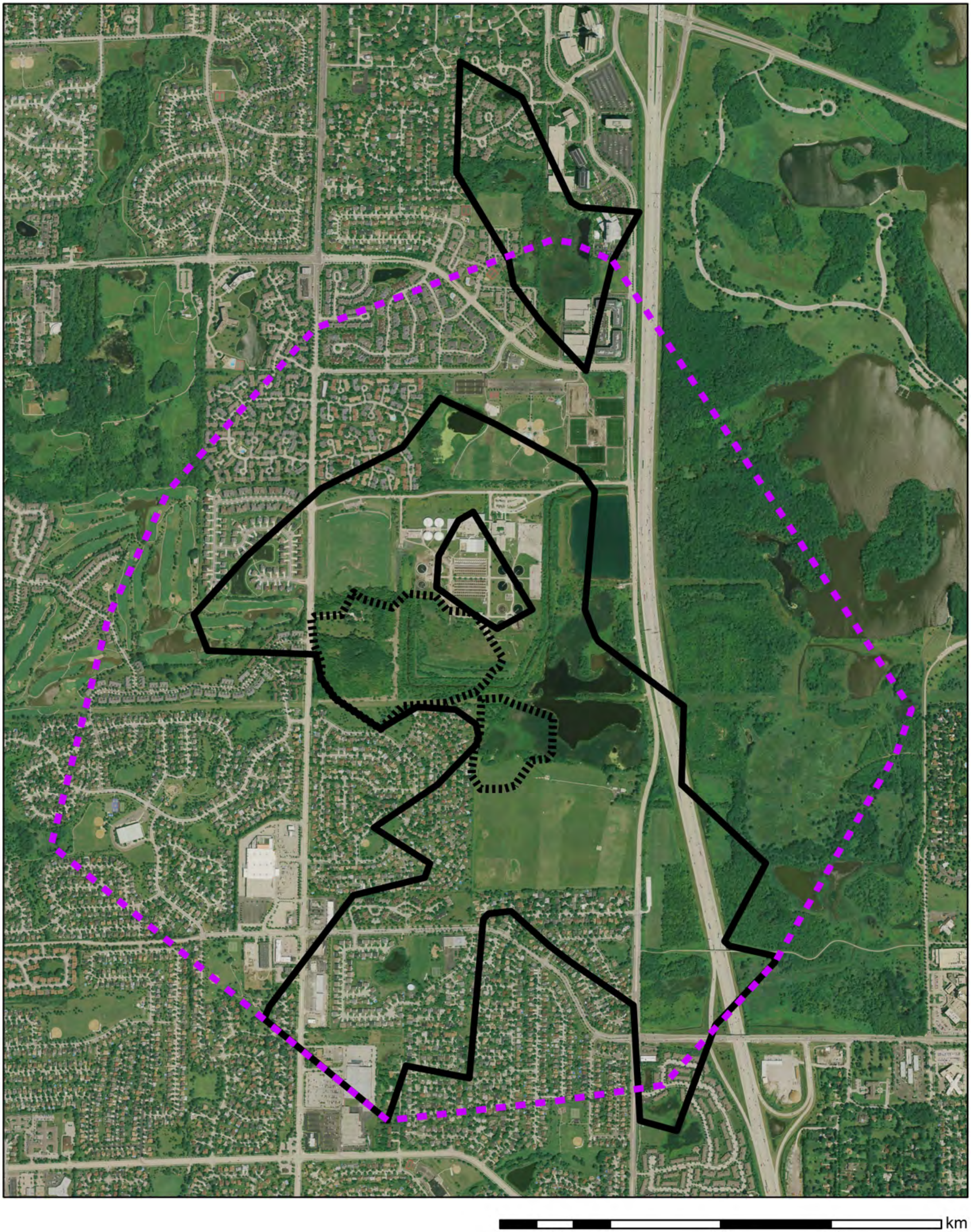

Figure 13

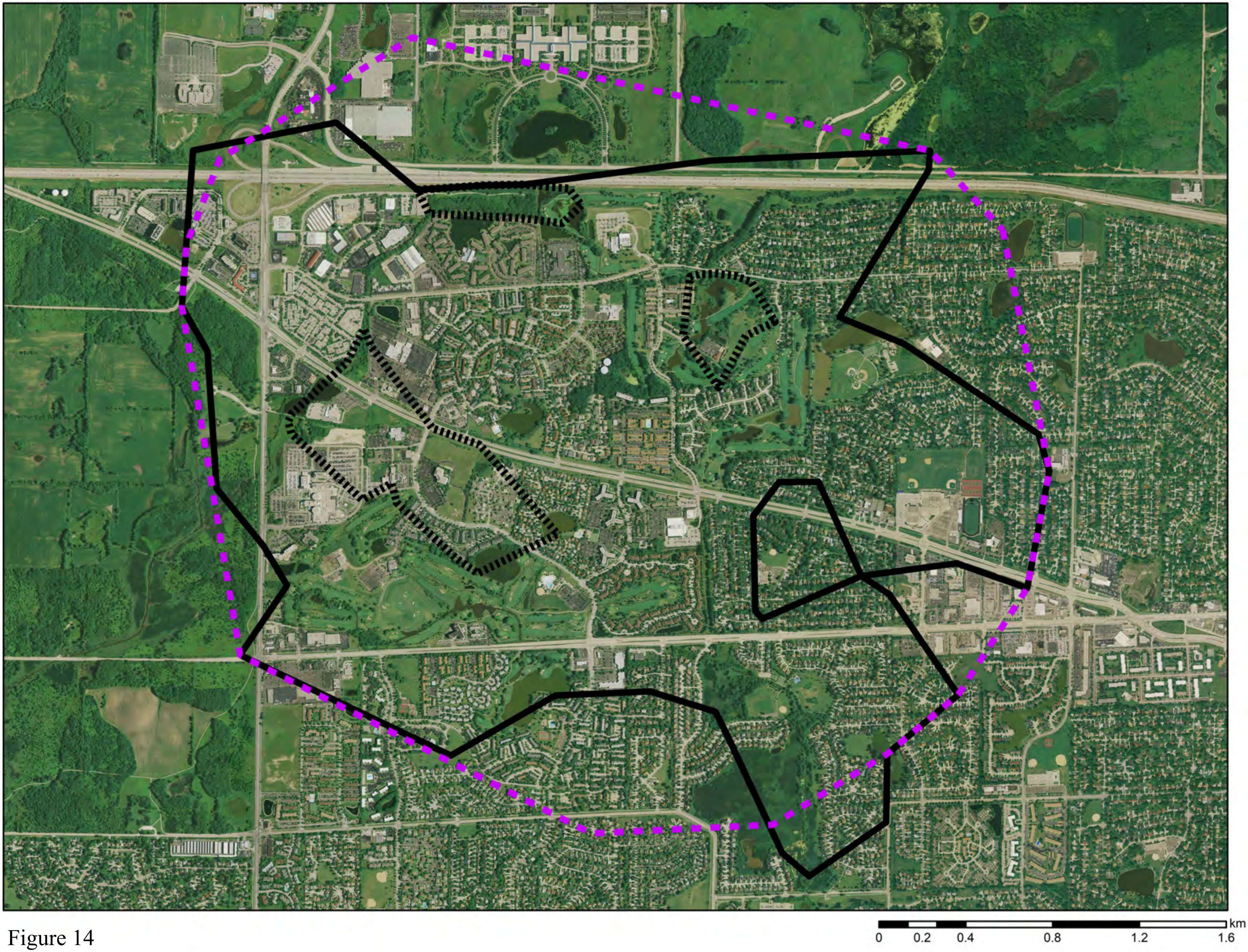

Figure 14

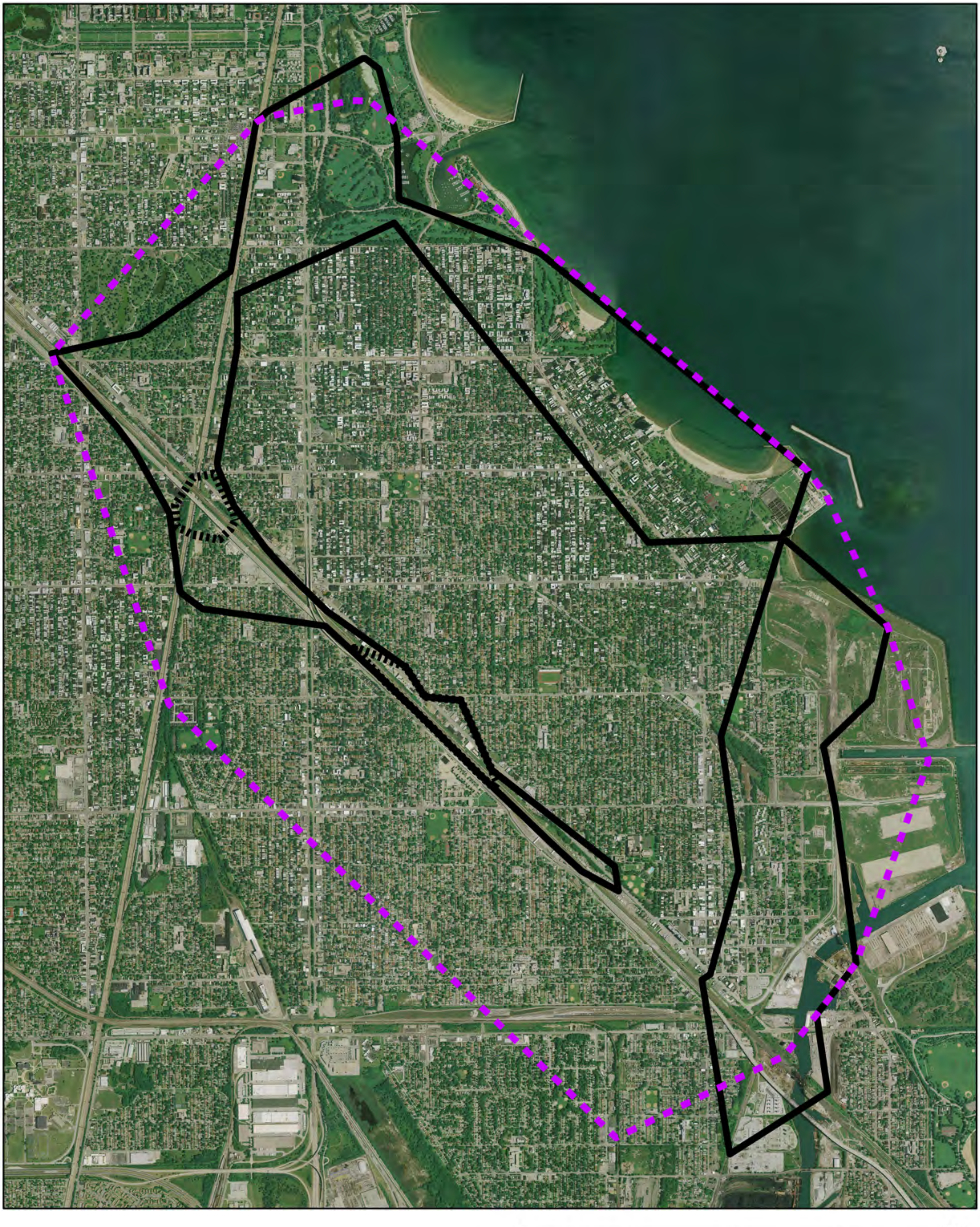

Figure 15

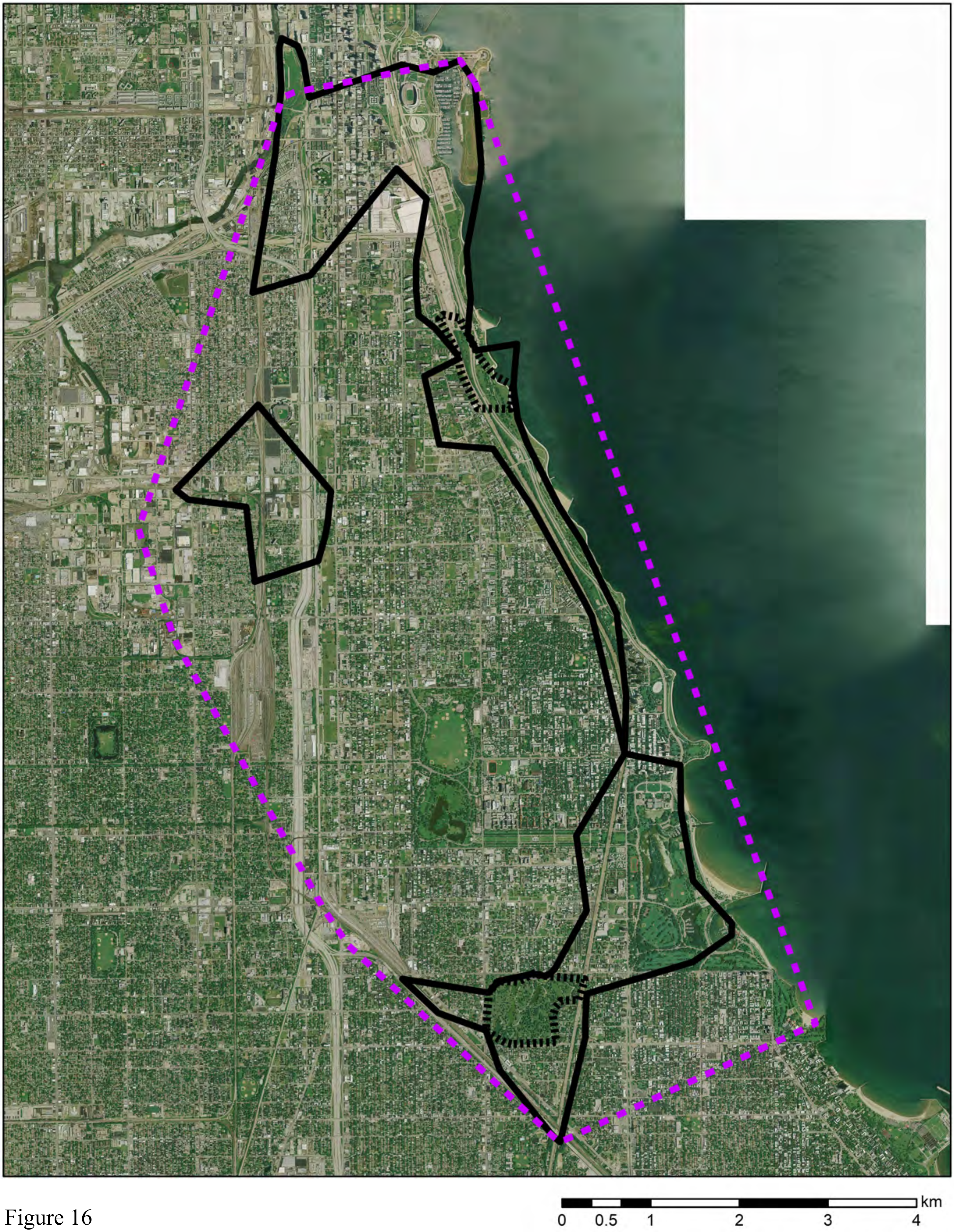

Figure 16

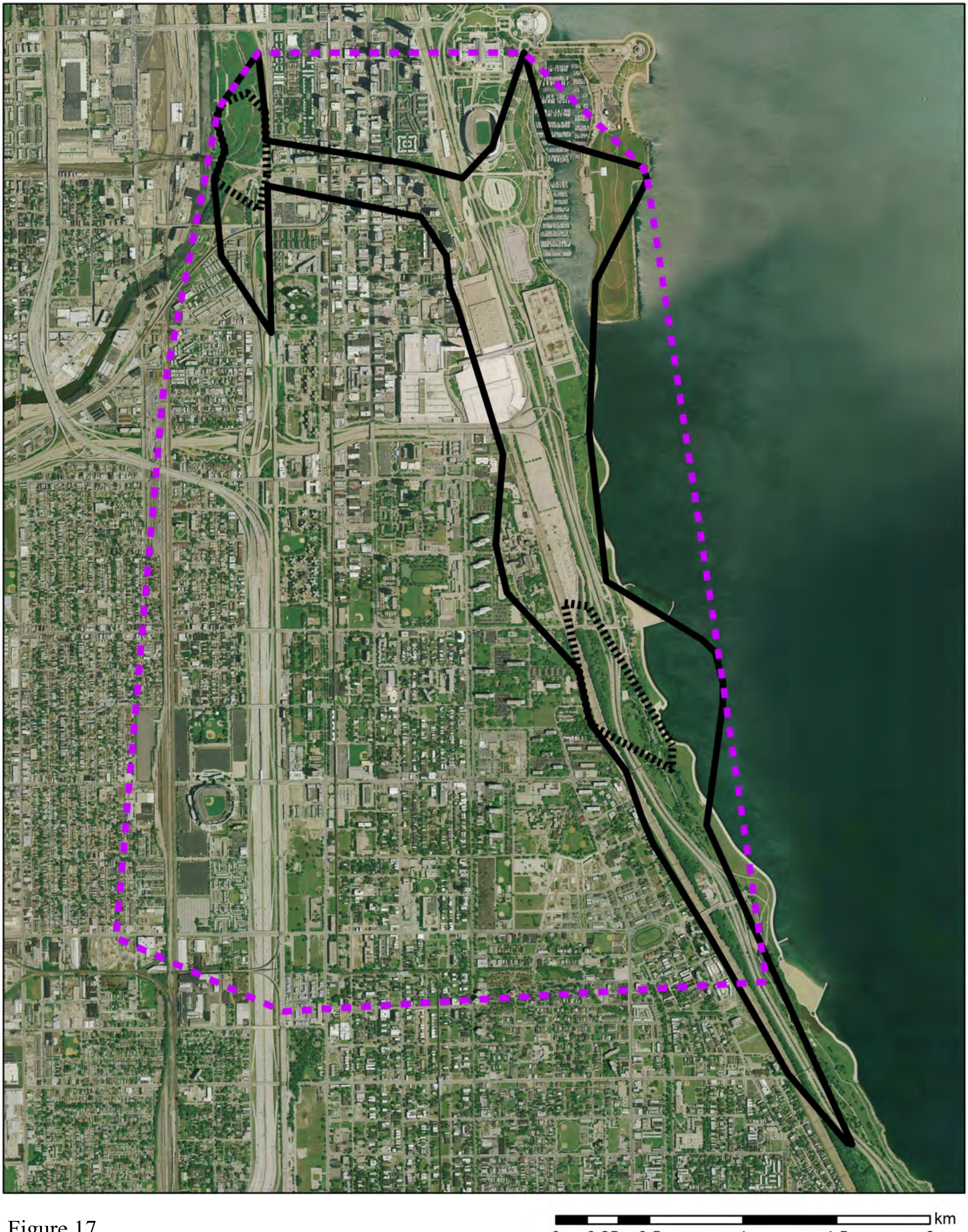

Figure 17

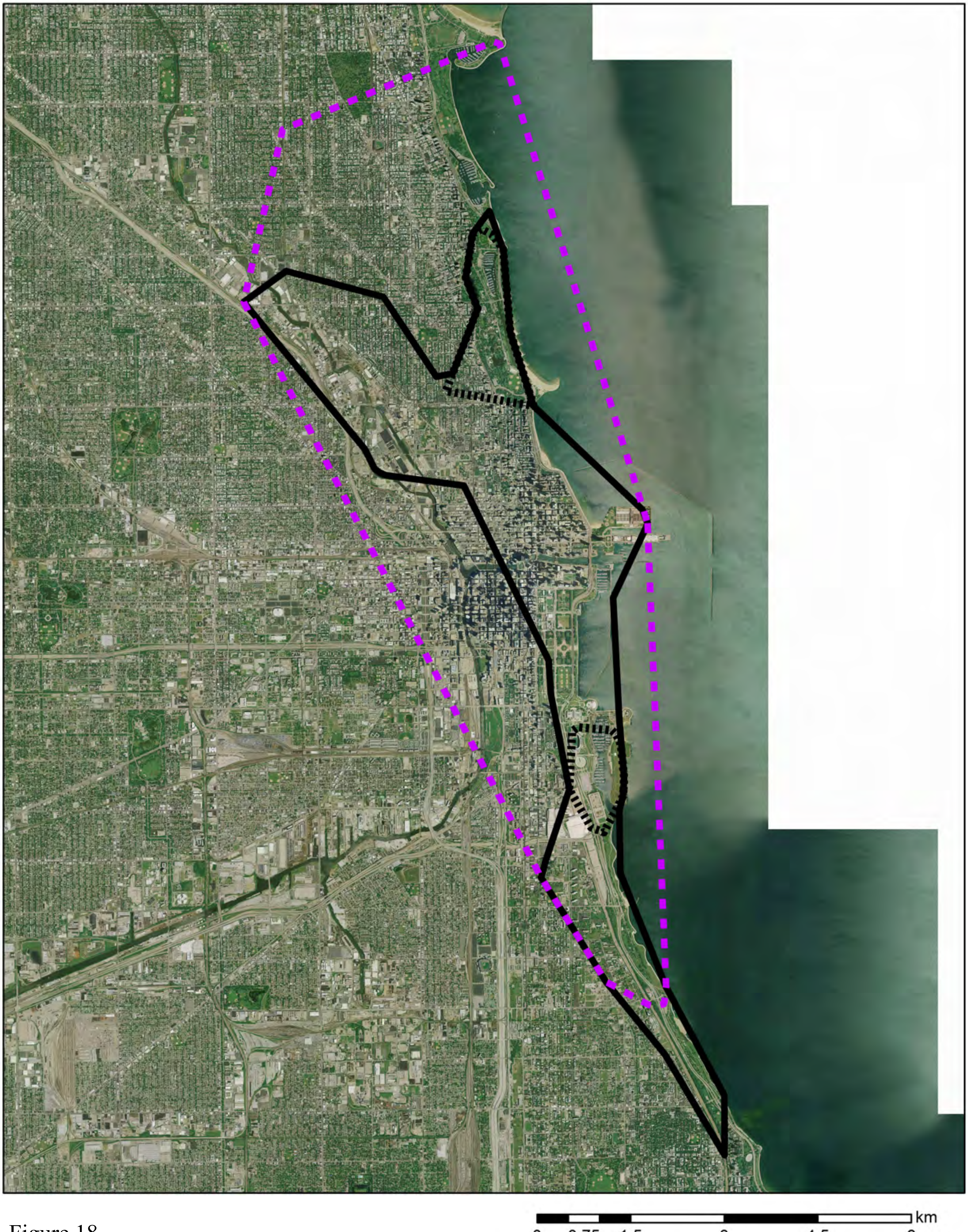

Figure 18

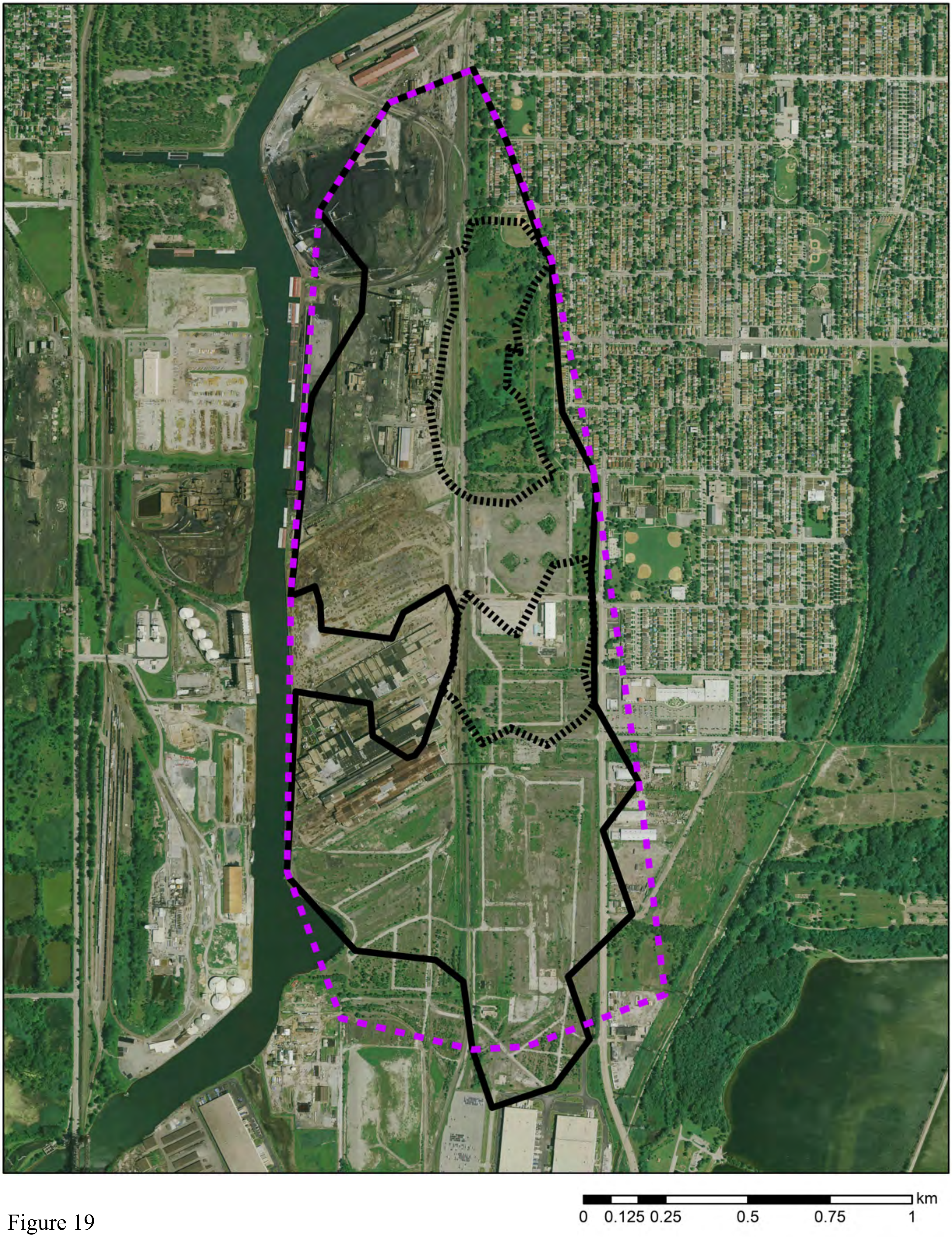

Figure 19

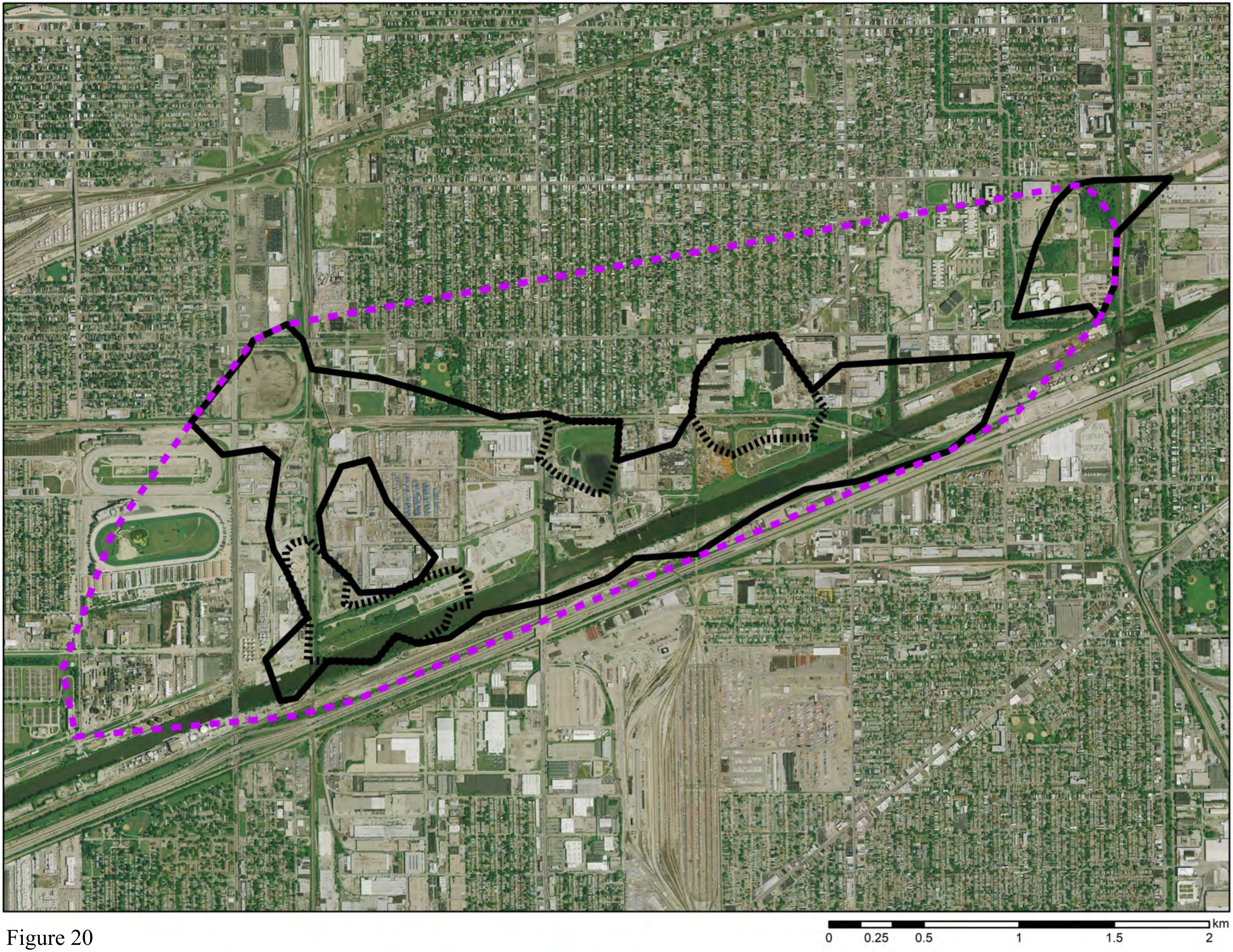

Figure 20

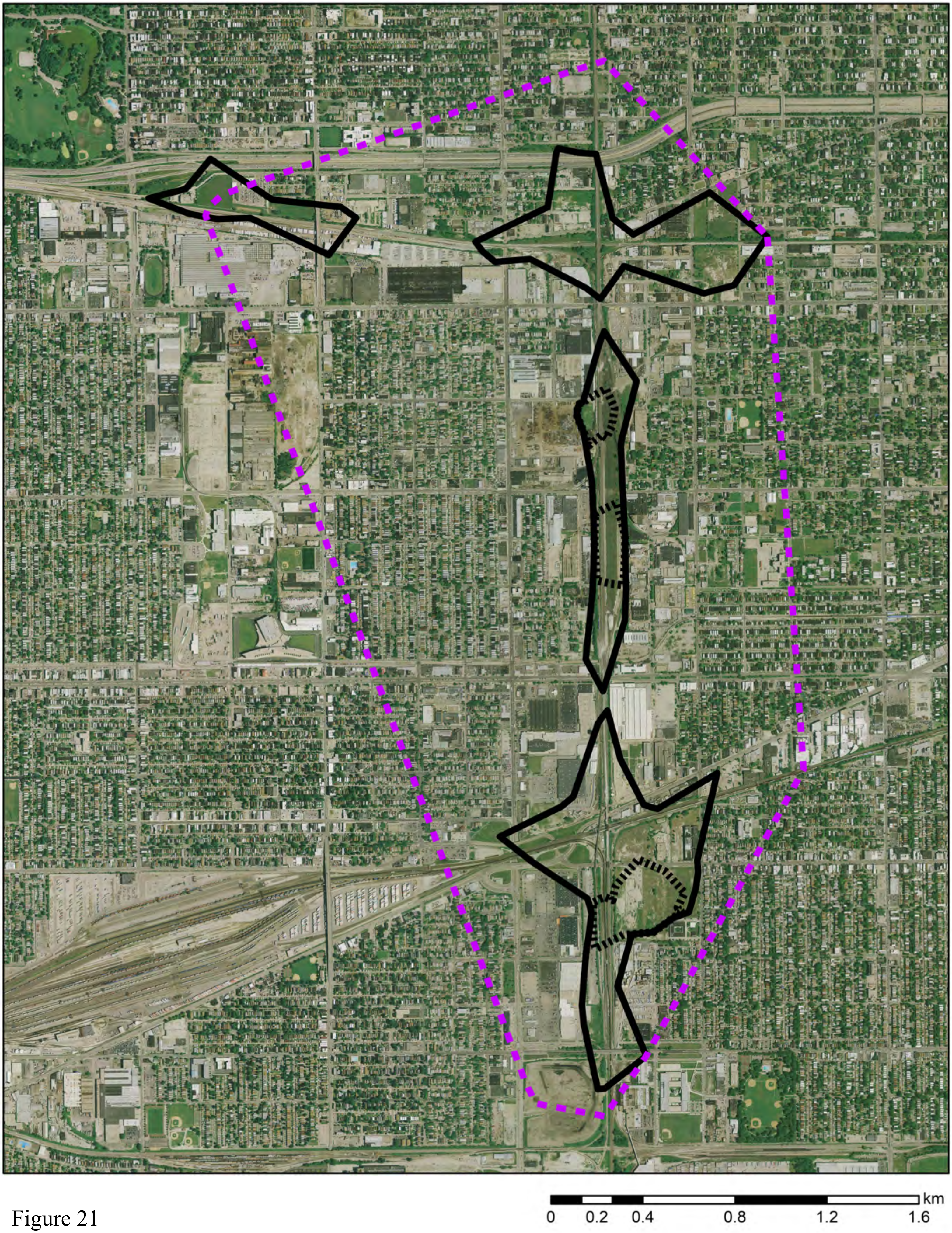

Figure 21

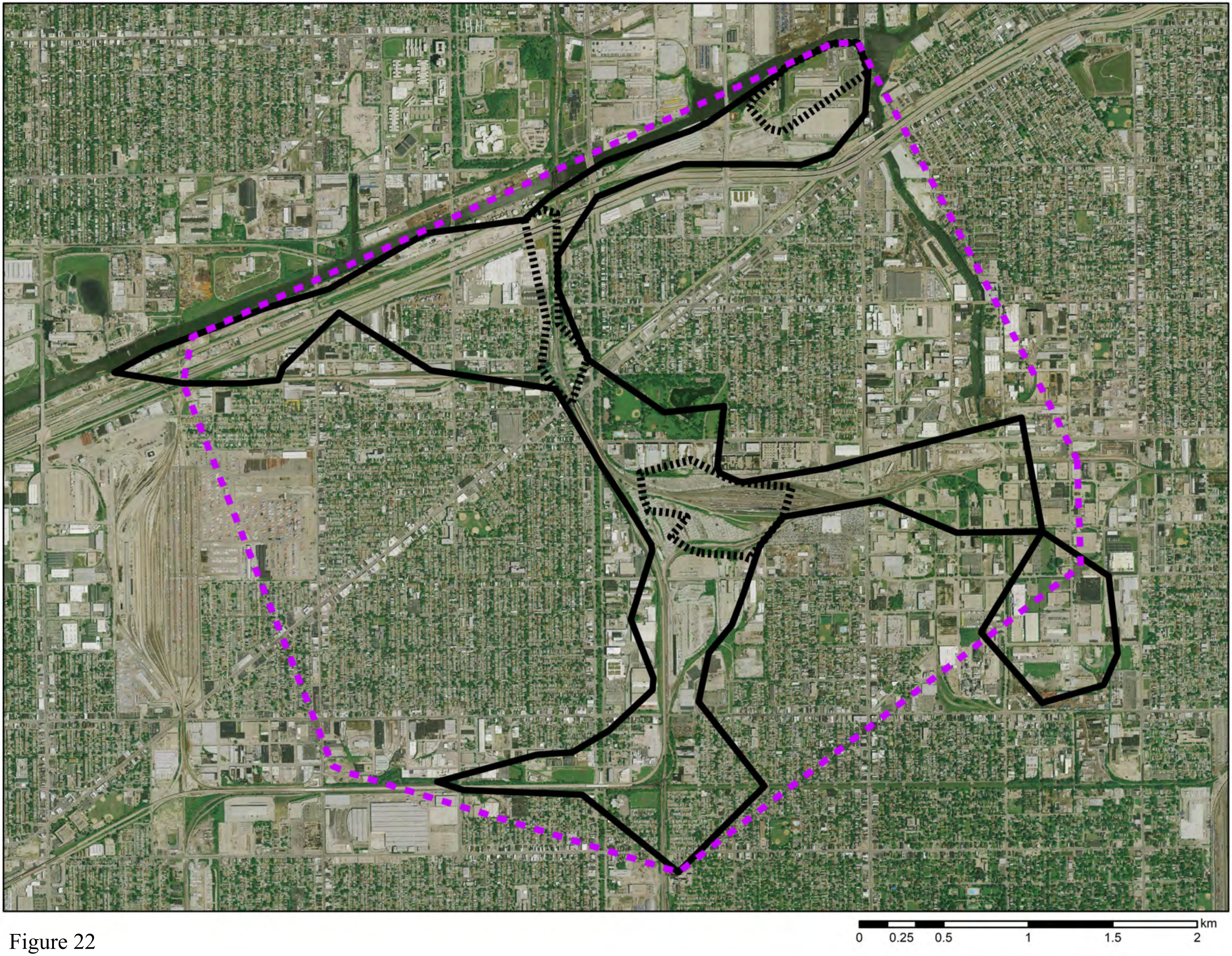

Figure 22

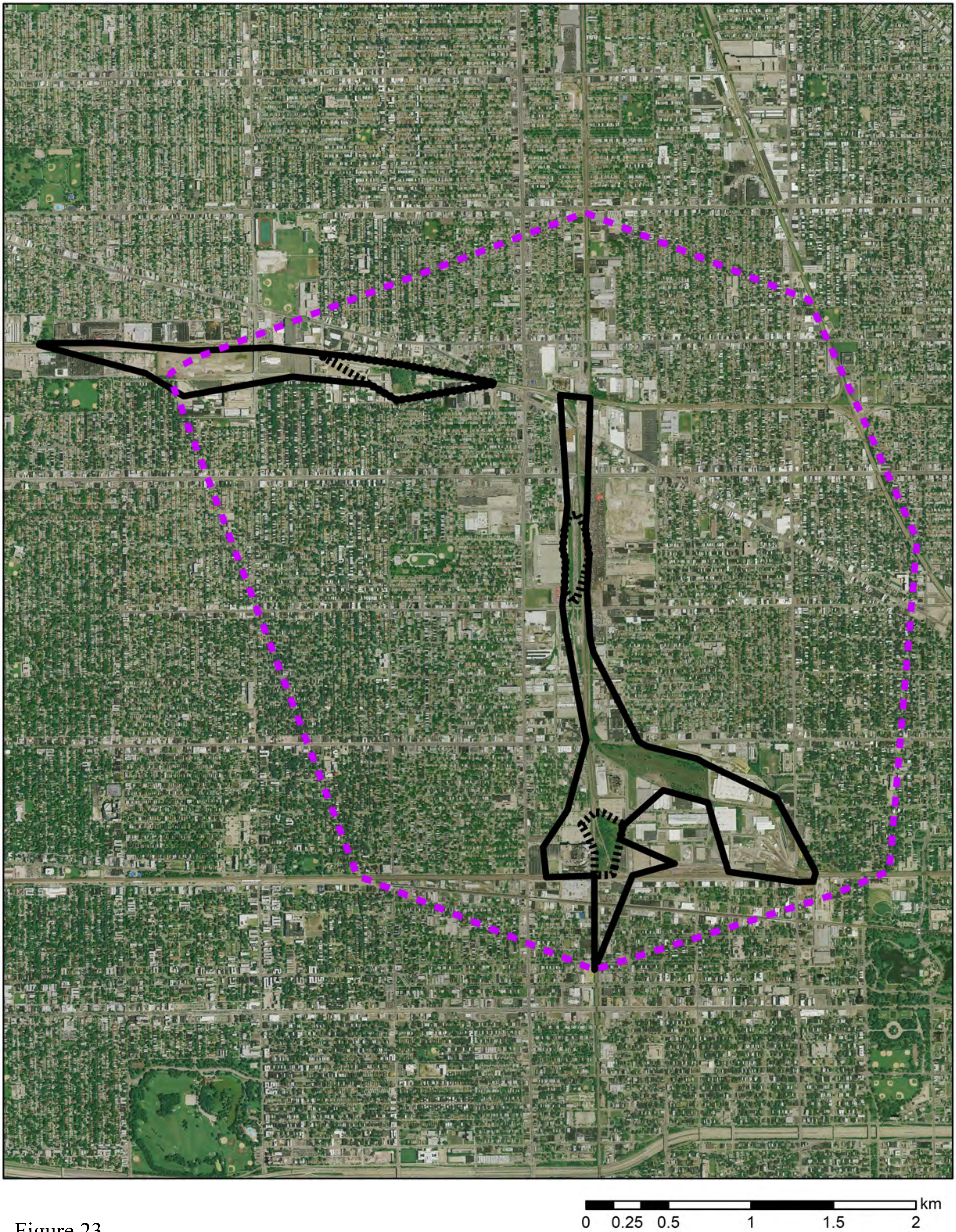

Figure 23
